# Availability of splicing factors in the nucleoplasm can regulate the release of mRNA from the gene after transcription

**DOI:** 10.1371/journal.pgen.1008459

**Published:** 2019-11-25

**Authors:** Hodaya Hochberg-Laufer, Noa Neufeld, Yehuda Brody, Shani Nadav-Eliyahu, Rakefet Ben-Yishay, Yaron Shav-Tal

**Affiliations:** The Mina & Everard Goodman Faculty of Life Sciences & Institute of Nanotechnology, Bar-Ilan University, Ramat Gan, Israel; University of Basel, SWITZERLAND

## Abstract

Gene expression dynamics can be measured in single living cells. Using a detectable transcriptionally active gene in living cells, we previously found that an mRNA undergoing several splicing events was retained at this gene after transcription until completion of mRNA processing. To determine the reason for this delay in release and whether mRNA retention on the gene might depend on splicing factor availability, we modulated the levels of splicing factors in the nucleus. Increasing the abundance of the diffusing fraction of splicing factors by their overexpression or by Clk1 kinase overexpression to disassemble nuclear speckles, led to a reduction in splicing factor residence times on the active gene, and the retained mRNA was rapidly released from the gene. Other treatments such as overexpression of a mutant inactive Clk1, the downregulation of MALAT1 lncRNA or of the Son protein, or the overexpression of the splicing factor import factor TNPO3, did not affect the dynamics of mRNA release from the gene. We found that the faster release of the mRNA from the gene mediated by increased availability of splicing factors, was dependent on the RS domain of the splicing factors and its phosphorylation state. We propose that the relative abundancies of splicing factors in the nucleoplasm can affect their availability for the splicing events taking place, and regulate the kinetics of mRNA release from the gene after processing.

## Author summary

Genetic information is contained in the cell nucleus and encodes proteins. However, protein production takes place in the cytoplasm, and so a molecule is needed to connect between the nucleus and cytoplasm. This messenger molecule is called messenger RNA (mRNA). It is produced and copied from the DNA, and after some processing will travel to the cytoplasm to encode proteins. This study focuses on the timing of mRNA release from the gene after it is copied from the DNA. Processing of mRNA includes the removal of some of its pieces and the stitching back of the remaining parts. This is called splicing. We found that mRNAs undergoing many splicing events are retained on the gene until splicing has completed, and examined what is the cause for this delay. We found that the factors performing the splicing might be limiting the process if their levels are not high enough at the gene locus. By increasing splicing factor levels in the nucleus we show that their abundance increases the rate at which mRNA is released. This means that the cell can regulate gene expression rates by limiting the availability of splicing factors that are free to take part in the processing of mRNA.

## Introduction

Transcription and pre-mRNA processing are orchestrated processes that can occur in parallel [1–3]. The cap structure is added to the pre-mRNA during transcription as are the many mRNA-binding proteins that assemble on the mRNA molecule to generate the mRNP structure [4, 5]. A large variety of splicing factors form the spliceosome and can co-transcriptionally interact with the pre-mRNA. Some introns are co-transcriptionally spliced while others are excised later on during the nucleoplasmic phase of the mRNA [6–9].

Splicing factors roam the nucleoplasm and are recruited to active genes to participate in splicing events required for the processing of the pre-mRNA. Imaging of splicing factors in intact cells shows that they also localize in 10–30 sub-nuclear structures currently coined “nuclear speckles” [10–14]. Much speculation and confusion regarding the function of these structures in transcription and pre-mRNA splicing has originated from the identification of RNA within. Together with the identification of an abundant population of splicing factors in nuclear speckles as well as kinases that specifically phosphorylate some of these factors, it was not far-reaching to assume some role for these structures in pre-mRNA processing [15]. Two recent studies examining genome structure and organization at high resolution have found that nuclear speckles tend to localize around genomic regions with high transcriptional activity [16, 17].

Some studies have suggested that nuclear speckles function as splicing factor storage and/or recycling depots [18]. In this model, splicing factors leave the nuclear speckle and travel the nucleoplasm to function in splicing on active genes. The “used” splicing factors must then return to the nuclear speckles where they are re-phosphorylated and stored until needed for the next cycle. The shuttling of splicing factors between nuclear speckles and the nucleoplasm was observed in the first photobleaching experiments performed in the nucleus of a GFP-tagged splicing factor (the ASF/SF2 SR protein, now termed SRSF1) [19, 20]. A maximal residence time of 50 sec in nuclear speckles was calculated by modeling the data [19]. A quantitative study performed on several GFP-tagged splicing factors in living cells showed that splicing factors constantly diffuse through the nucleoplasm and nuclear speckles even when splicing is inhibited [21]. Mathematical modeling of the data suggested that continuous movement and random transient interactions with pre-mRNA take place without the need for a storage capacity in the speckles. In another quantitative live-cell imaging study, the assembly of splicing factors on pre-mRNAs was measured showing that interactions lasted for up to 30 sec suggesting that splicing is accomplished within this time frame [22], as was later shown by direct measurements in living cells [23].

Previously, we followed the transcription and release of mRNAs that undergo splicing on active genes in living cells, by examining the kinetics of mRNA transcription in real time [24]. We found that an mRNA undergoing many splicing events was retained on the gene after transcription for substantially longer time periods, compared to a similar mRNA that underwent fewer splicing events and was released almost immediately after transcription. We determined that the lag in the release of the retained mRNA was not due to changes in transcription elongation. Rather, the effect was splicing dependent. Now, we wanted to determine the reason splicing was connected to this delay in the release of the mRNA from the gene. Since the lag was related to splicing, we hypothesized that varying the nucleoplasmic abundance of splicing factors that can also regularly accumulate in nuclear speckles, might modify the lag between the end of transcription and the release of the mRNA from the gene. We used different approaches to increase the availability of splicing factors in the nucleoplasm, and then measured the dynamics of splicing factors that were associated with actively transcribing genes and the release rate of the transcribed mRNA. Our results demonstrate that the abundance and availability of active splicing factors in the nucleoplasm for participation in the splicing process can influence the rate at which mRNAs are released from the gene after transcription.

## Results

### Measuring the dynamics of splicing factors on an active gene

**Fig 1:**
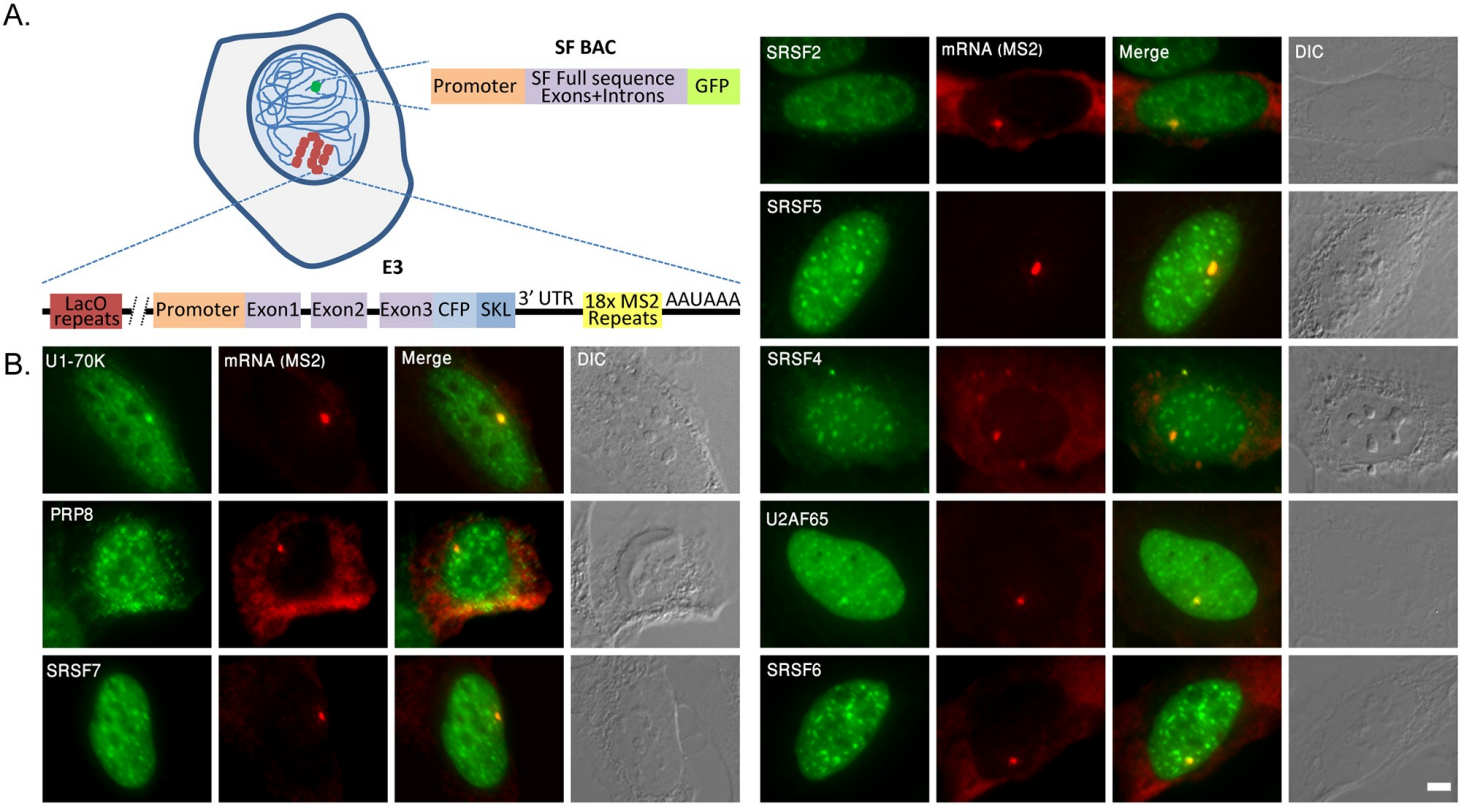
Splicing factors are recruited to an actively transcribing gene. (A) The cell system consists of U2OS cells that contain one type of a stably integrated GFP-tagged splicing factor BAC as well as a β-globin mini-gene termed E3, co-integrated with *lacO* repeats for the detection of the genomic locus. The mRNAs transcribed from this gene can be detected by the MS2 repeats appearing in the 3’UTR, and the protein translated from the mRNA is targeted to cytoplasmic peroxisomes by a CFP-SKL sequence. (B) GFP-tagged splicing factors expressed from the different BACs (green) appear in the nucleoplasm, in nuclear speckles and are recruited to the active transcription site of the E3 gene that is detected using RNA FISH with a probe that hybridizes with the MS2 repeats (red). DIC in grey. Bar = 5 μm.

To test the effect of splicing factor abundance in the nucleoplasm on the kinetics of the release of a spliced mRNA from a gene after its transcription, we first characterized the real-time interactions of splicing factors with an active gene in living cells, using a cell system [24] that allows the real-time detection of an inducible gene array [25]. This cell system contains a stably integrated Tet-inducible β-globin mini-gene termed E3 in U2OS cells (Fig 1A). The coding sequence has 3 exons and 2 introns, and then includes an in-frame cyan fluorescent protein sequence with a SKL peroxisome-targeting peptide (CFP-SKL). This allows the detection of the translated protein in cyan-labeled cytoplasmic peroxisomes. The gene is induced by adding doxycycline (dox) to the medium. To detect the mRNA transcribed from the gene, the 3’-untranslated region (3’UTR) contains 18 repeats of the MS2-binding sites. The mRNA can be detected in fixed cells by RNA FISH with fluorescent probes that hybridize to the MS2 repeats, or in living cells with a fluorescently tagged MS2-coat protein (FP-MS2-CP) that binds to the repeated stem-loop structure of the MS2 repeats in the mRNA. Finally, the genomic locus of integration is detected with a fluorescently tagged lac repressor protein (FP-LacI) that binds to lac operator repeats (*lacO*) co-integrated with the gene, and so the gene can be detected also in an inactive state.

Previously, the recruitment of endogenous snRNPs and SR proteins to the E3 gene array observed when the gene was induced to transcribe using RNA FISH and immunofluorescence in fixed cells, showed that co-transcriptional splicing was taking place on the gene [24]. The splicing factors were not recruited to similar genes that do not contain introns, and so are unspliced, meaning that most of the splicing factors that are associated with the gene locus are present there due to the specific pre-mRNA that is being transcribed there. Now, in this study we set out to measure the recruitment dynamics of splicing factors in living cells by expressing GFP-tagged splicing factors suitable for live-cell studies. In order to avoid overexpression of splicing factors from plasmids with viral promoters, we stably integrated bacterial artificial chromosomes (BACs) containing the full gene body of several splicing factors under the control of their endogenous promoters with an in-frame GFP-tag [26]. Thus, the BACs transcribe GFP-fused splicing factors at endogenous levels and under physiological regulation, thereby not overloading the cells with excess proteins [27].

The following GFP-tagged splicing factor BACs were integrated into the E3 cells. SR proteins: SRSF2 (SC35), SRSF4 (SRp75), SRSF5 (SRp40), SRSF6 (SRp55) and SRSF7 (9G8); snRNP components: U1-70K (part of the U1 snRNP, which binds to the 5’-splice site), PRP8 (part of the U5 snRNP, which is part of the U4/U6.U5 triple-snRNP); and U2AF65 (U2 snRNP auxiliary factor, which binds to the polypyrimidine tract). As expected, all the GFP-tagged splicing factors showed prominent presence in nuclear speckles and nucleoplasmic distribution (Fig 1B). In addition, recruitment and accumulation of the splicing factors on the active E3 gene was observed (under dox induction). This particular accumulation resembled other nuclear speckles but could usually be distinguished from them due to higher intensity levels on the active gene (Fig 1B).

**Fig 2:**
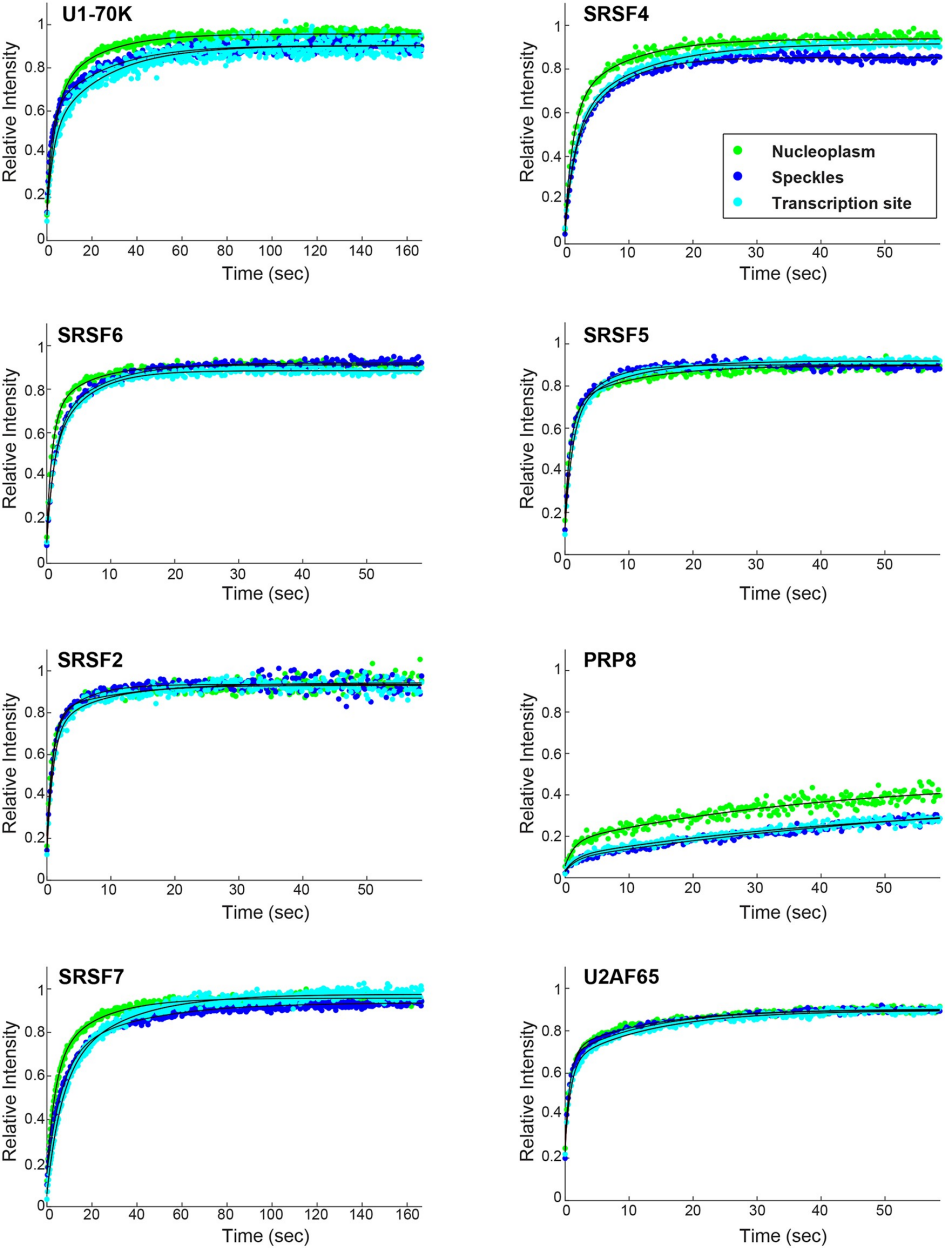
The intra-nuclear dynamics of splicing factors in different nuclear compartments can vary. Recovery curves of FRAP measurements performed on the splicing factors U1-70K, SRSF4, SRSF6, SRSF5, SRSF2, PRP8, SRSF7 and U2AF65 taken in the nucleoplasm (green), in nuclear speckles (blue) and at the transcription site (cyan). The relative intensity of each plot represents at least 10 experiments that were performed on 3 independent days. The statistical differences between the experiments and the relative fractions are presented in S1 and S2 Tables, respectively.

The dynamics of the different splicing factors were measured in three sub-nuclear compartments using a fluorescence recovery after photobleaching (FRAP) approach: on the active gene, in the nucleoplasm, and in nuclear speckles (Fig 2). Curve fitting of the recovery plots showed that all were best fitted with two exponents, implying the existence of at least two sub-populations with different kinetic profiles, namely, an unbound rapidly diffusing sub-population and a bound fraction. Comparing the dynamic behavior of each factor between the three compartments showed that some factors had identical dynamics in all regions (SRSF2, SRSF5, SRSF6, U2AF65), whereas some factors had distinctly different dynamics in each compartment (U1-70K, SRSF7, PRP8, SRSF4) (S1 and S2 Tables).

The above measurements show that splicing factor dynamics are different in nuclear speckles compared to the factors assembled on the active gene, and might imply that such measurements can discern between splicing factors that are engaged in co-transcriptional splicing on an active gene versus nucleoplasmic assemblies of non-active splicing factors in nuclear speckles. These results may also imply that different splicing factors can differentially affect processing rates.

### Overexpression of several splicing factors can modulate the release rate of mRNA from the gene

Next, we were interested in determining the reason for the delay in the release of a spliced mRNA from a gene after transcription. Examination of the release rate of the mRNA from the gene in living cells is performed using FRAP, while in this case the nascent mRNAs being transcribed on the gene are photobleached, as we previously described [24]. The E3 mRNA described above contains MS2 sequence repeats in its 3’UTR, which are bound by YFP-MS2-CPs such that the active gene can be seen in living cells (S1A Fig). These YFP-MS2-CP’s are the proteins that are photobleached and FRAP recovery curves are obtained (S1B Fig). The E3 gene and other genes tested gave a certain FRAP curve. Importantly for the current assay of mRNA release kinetics, in this previous study we found that a larger version of the mini-gene we were using, termed E6 and containing six exons and five introns, hence having more splicing events, showed very different mRNA FRAP recovery kinetics. Specifically, mRNA kinetics on the E6 gene showed a significantly slower FRAP recovery curve in comparison to the E3 gene (S1B Fig). Using experiments in live and fixed cells together with simulations of the data, we previously demonstrated that the slow FRAP recovery curve is due to a prominent delay in the release time of the spliced E6 transcripts from the gene compared to E3 transcripts that were not retained on the gene [24]. We showed that this delay was not due to modulation of transcription elongation kinetics. Rather, when splicing was inhibited, the E6 FRAP curve no longer showed slow recovery, and was identical to the E3 curve, meaning that under these conditions there was no delay in the release of the E6 pre-mRNA from the gene, and that the delay effect is splicing dependent.

**Fig 3:**
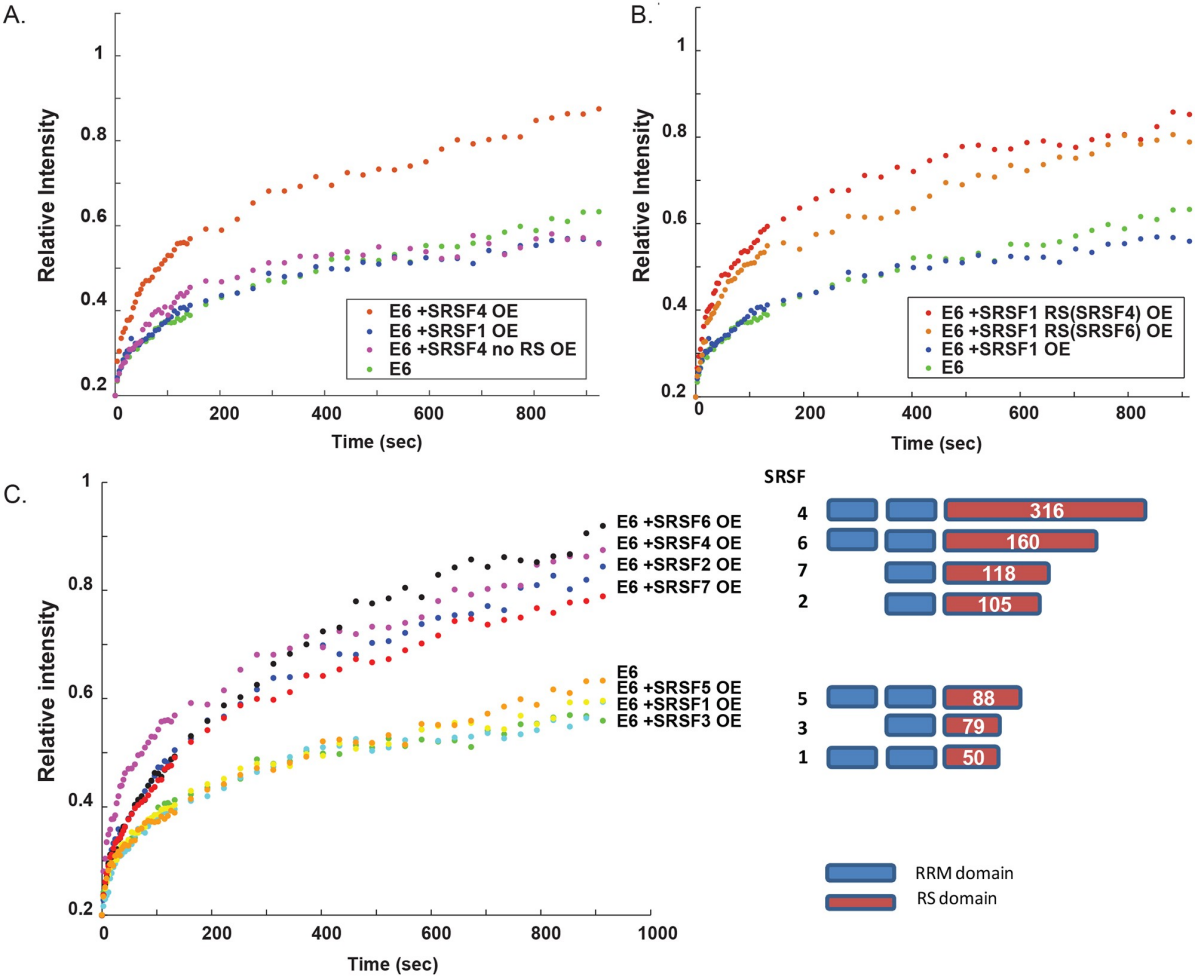
Overexpression of some SR proteins can modulate the rates of the E6 FRAP recovery curves. (A) Recovery curves of the YFP-MS2 mRNA FRAP measurements performed on the E6 transcription sites after the overexpression of SRSF1, SRSF4 or a truncated SRSF4 lacking its RS domain. The relative intensity of each plot represents at least 10 experiments that were performed on 3 independent days. (B) Recovery curves of the YFP-MS2 mRNA FRAP measurements performed on the E6 transcription sites after the overexpression of SRSF1 or a chimeric SRSF1 containing the RS domains of either SRSF4 or SRSF6. (C) Recovery curves of the YFP-MS2 mRNA FRAP measurements performed on the E6 transcription sites after the overexpression of the SR proteins SRSF1 to SRSF7. The scheme (right) depicts the structural organization of the SR proteins SRSF1-7. Each factor has one or two RNA recognition motifs (RRM, blue) and different sizes of the RS domain containing the serine and arginine repeats (RS domain, red). The statistical differences between the experiments are presented in S3 Table. Recovery curves with error bars appear in S2A–S2C Fig.

Now, we hypothesized that the relative availability of nucleoplasmic splicing factors en route for splicing, might limit or enhance the release rates of the mRNA. To test whether the availability of several splicing factors can regulate the release of mRNA from the gene, we chose at first to overexpress two SR proteins that differ in the size of their RS domains–SRSF4 has a large RS domain and SRSF1 has a small RS domain. We found that when SRSF4 was overexpressed, the slow E6 mRNA FRAP curve reverted to a fast recovery curve (like E3 i.e. no delay in mRNA release from the gene), whereas SRSF1 overexpression had no effect on the dynamics (Fig 3A; FRAP curves with error bars appear in S2 Fig). When the RS domain from SRSF4 was deleted, the truncated protein could still enter the nucleus (S3 Fig), but the E6 mRNA FRAP curve remained with slow dynamics (Fig 3A), showing that the RS domain is important for the splicing effect and the faster release of the E6 mRNA from the gene. Exchanging the RS domain of SRSF1 with the RS domain of SRSF4, led the chimeric SRSF1(+RS SRSF4) to change the E6 mRNA FRAP curve to faster kinetics (Fig 3B). Adding a different elaborate RS domain of SRSF6 to SRSF1 also has this effect (Fig 3B). Subsequently, we overexpressed a whole set of SR proteins and found that they fell into two categories–those that did not have any effect on the FRAP curves had small RS domains while the splicing factors with large RS domains had an effect, such that the E6 mRNA slow FRAP recovery plot reverted to faster recovery (Fig 3C, S3 Table). These results demonstrate that changing the relative amounts of certain splicing factors in the nucleoplasm by overexpression has a clear effect on whether there is a delay or not in the release of the mRNA from the gene. This implies that there is an internal balance between pools of splicing factors that are either available or unavailable for splicing, and this might be regulated by the relative amounts of splicing factors that are within nuclear speckles to those that are in the nucleoplasm. This hypothesis was tested below.

### Nucleoplasmic dispersion of endogenous splicing factors affects the release of mRNA from the gene

**Fig 4:**
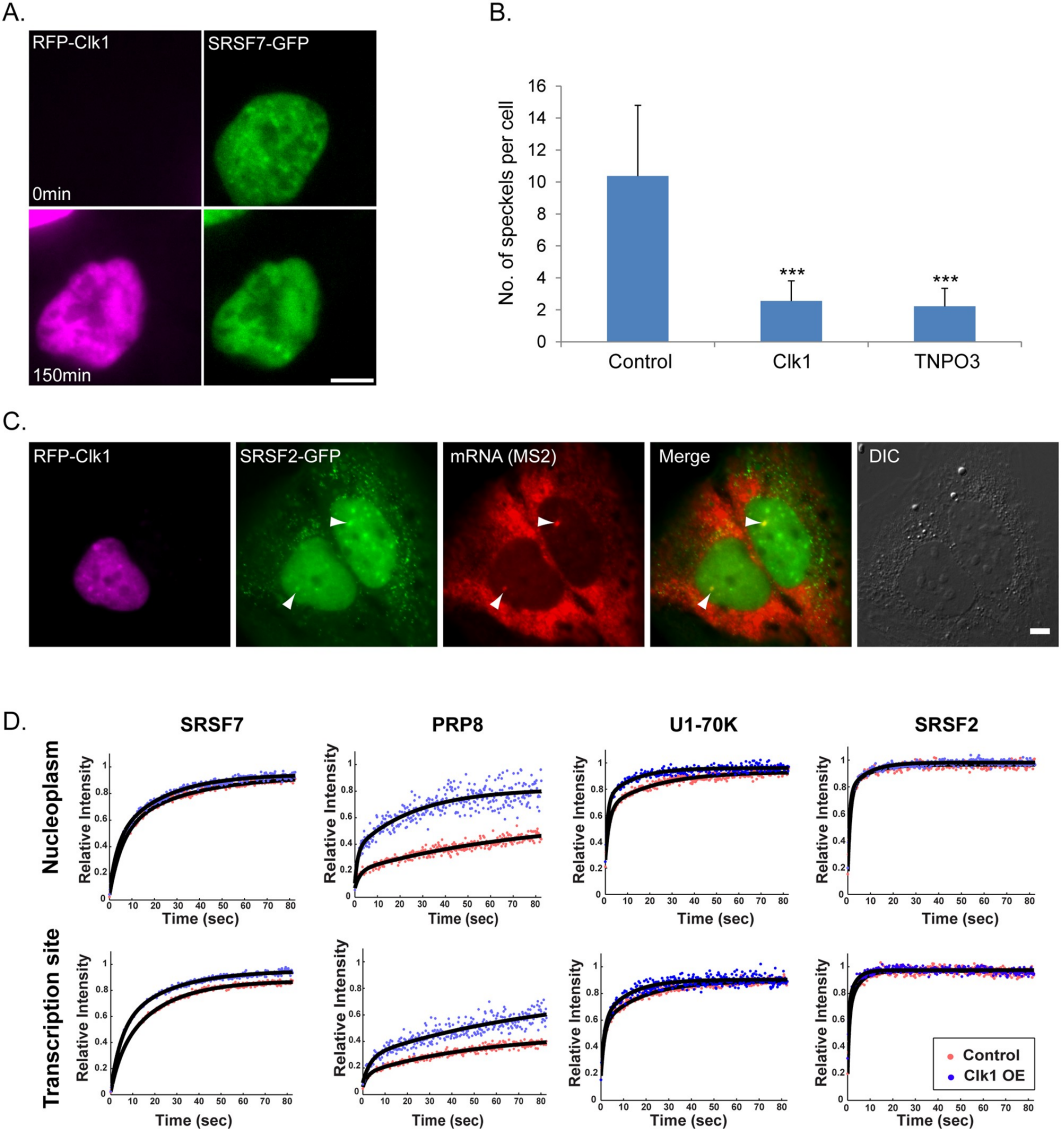
Clk1 overexpression does not prevent the recruitment of splicing factors to the transcription site and increases their intra-nuclear dynamics. (A) Nuclear speckles observed by SRSF7-GFP (green) begin to disperse as RFP-Clk1 (magenta) expression increases. Frames from S1 Movie. (B) The number of nuclear speckles was counted in untreated cells, in Clk1 overexpressing and TNPO3 overexpressing cells. n = 30 cells, control; 36 cells, CLK1; 32 cells, TNPO3; ***p<0.001. (C) The recruitment of SRSF2 (green) to active transcription sites detected using RNA FISH with an MS2 probe (red) was examined in normal and in cells overexpressing Clk1 (magenta). DIC in grey. Bar = 5 μm. (D) Recovery curves of FRAP measurements performed on the splicing factors SRSF7, PRP8, U1-70K and SRSF2 taken in the nucleoplasm and at the transcription sites. In cells over-expressing Clk1 (blue), the splicing factor mobile fraction was usually more rapid than in control cells (red), seen by the shift in the curve in the Y axis. The relative intensity of each plot represents at least 10 experiments that were performed on 3 independent days. The statistical differences between the experiments are presented in S4 Table.

In order to increase the availability of all splicing factors without the use of splicing factor overexpression, we increased the nucleoplasmic abundance of splicing factors by dismantling nuclear speckles. The overexpression of the Clk1/STY kinase, responsible for the serine phosphorylations on the RS domain in SR proteins, causes the disassembly of nuclear speckles [28, 29]. We first determined that overexpression of RFP-Clk1 and the lack of nuclear speckles (Fig 4A and 4B and S1 Movie) did not interfere with the transcriptional activity of the gene. Indeed, the gene was active and splicing factors continued to accumulate on it (Fig 4C).

To examine whether the dispersion of endogenous splicing factors in the nucleoplasm can affect their general dynamics as measured above, we focused our analysis on factors that showed different dynamics in the FRAP measurements (Fig 2), namely, SRSF2 that showed rapid dynamics, SRSF7 and U1-70K that had intermediate dynamics, and PRP8 that was the slowest of them all. FRAP analysis of the dynamics of the splicing factors on the active gene and in the nucleoplasm under Clk1 overexpression conditions showed minor but significant differences in the dynamics for U1-70K, PRP8 and SRSF7 (Fig 4D, S4 Table). Altogether, these results suggest that the higher abundance of splicing factors in the nucleoplasm leads to more rapid interactions with the active gene (faster FRAP recovery curves). This in turn, might affect the rates at which the spliced mRNA is released from the gene.

**Fig 5:**
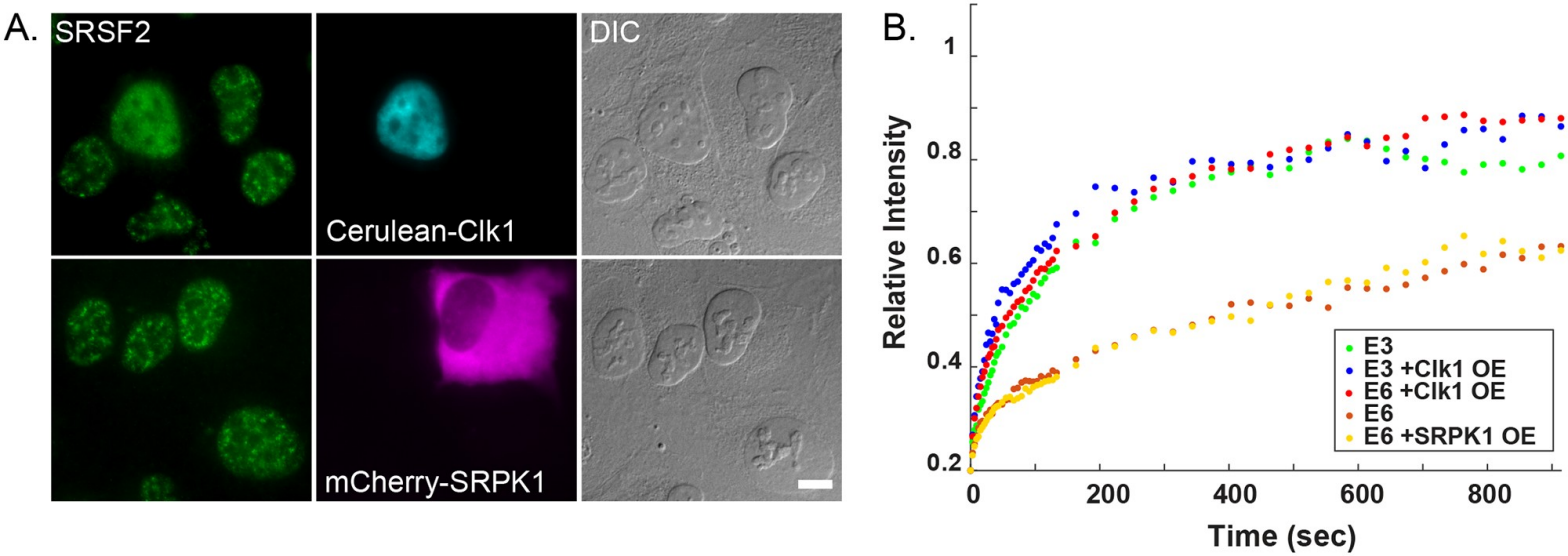
Overexpression of Clk1 but not SRPK1 leads to splicing factor dispersal and increases the FRAP recovery rates on the E6 gene. (A) The distribution of SRSF2 (green) in cells transfected with Cerulean-Clk1 (cyan) or mCherry-SRPK1 (magenta). DIC in grey. Bar = 5 μm. (B) Recovery curves of the YFP-MS2 mRNA FRAP measurements performed on the E3 and E6 transcription sites under normal conditions and after the overexpression of Clk1 or SRPK1. The relative intensity of each plot represents at least 10 experiments that were performed on 3 independent days. There was a more significant difference in the FRAP recovery rates on the E6 gene than the E3 gene in cells overexpressing Clk1 compared to control cells (One way ANOVA, p<0.0001, p = 0.029). The difference in the FRAP recovery rates on E6 gene overexpressing SRPK1 compared to control cells was not significant (One way ANOVA, p = 0.92029). Recovery curves with error bars appear in S2D Fig.

To test this, we overexpressed Cerulean-Clk1 in cells expressing the E3 or E6 genes, and analyzed the FRAP recovery curves of the YFP-MS2-CP coated mRNAs on the active genes (Fig 5A and 5B). Indeed, the slow recovery curve coming from the E6 gene was reverted to a fast recovery curve (like the E3 gene) when nuclear speckles disassembled and the splicing factors were dispersed in the nucleoplasm, meaning that there was no delay in the E6 transcript release from the gene. Clk1 overexpression had a minor effect on the FRAP curve of the E3 gene. The effect was specific to the Clk1 kinase and not to another kinase, SRPK1, which also phosphorylates splicing factors but does not release the splicing factors from nuclear speckles (Fig 5A and 5B). Altogether, these results suggest that under regular conditions a sub-population of splicing factors are contained in nuclear speckles and are not available for the splicing reactions on the active gene. The dismantling of nuclear speckles changes this balance and more splicing factors are available, such that splicing proceeds more efficiently and the E6 mRNA can be released without delay.

To test whether the mere alteration in the distribution of the splicing factors from nuclear speckles is responsible for affecting the release of the mRNA from the gene, we knocked down the levels of Son by siRNA treatment. Son is a nuclear speckle component [30], and its reduction leads to the formation of doughnut-shaped nuclear speckles containing less splicing factors confined to the middle of the structure (S4A and S4B Fig). This treatment did not affect the transcription FRAP curves on the E3 and E6 genes (S4C Fig). Also, the accumulations of splicing factors on the genes were not affected. For instance, the SRSF7 splicing factor continued to accumulate on the active gene (S4D Fig), as also described for U1-70K and SRSF1 [31].

Another nuclear speckle structural factor that we tested was the lncRNA MALAT1. This molecule was not enriched on the E6 active gene under regular conditions and nor when splicing factors were dispersed in the nucleoplasm by Clk1 overexpression (S5A Fig). We knocked out the *MALAT1* gene using CRISPR-Cas9 (S5B and S5C Fig) and did not see a change in the E6 FRAP curve (S5D Fig), nor in the recruitment of splicing factors to the active gene (S6 Fig).

**Fig 6:**
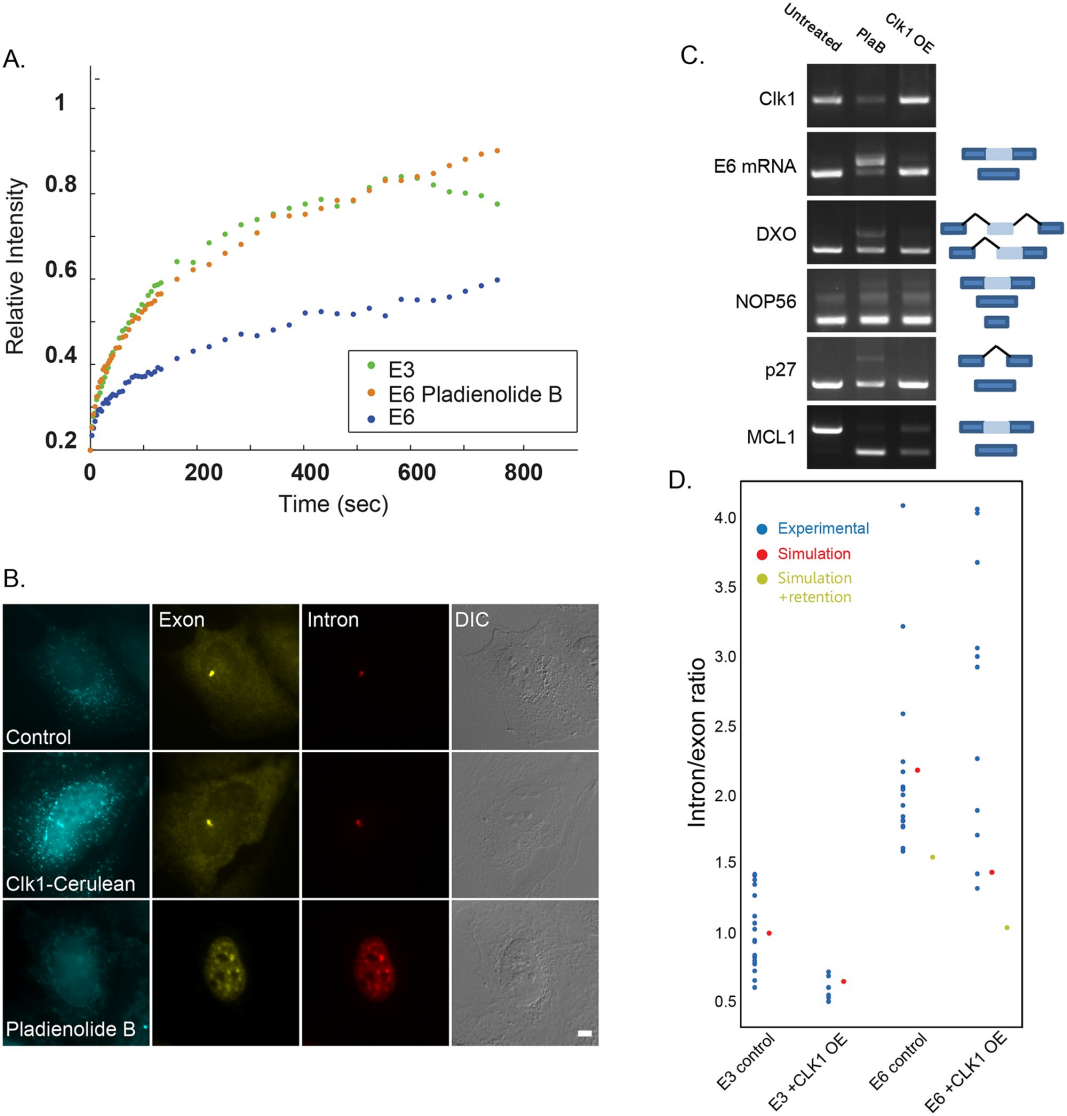
Clk1 overexpression does not inhibit splicing. (A) Recovery curves of the YFP-MS2 mRNA FRAP measurements performed on the E3 and E6 transcription sites under normal conditions and after treatment with the splicing inhibitor Pladienolide B. The relative intensity of each plot represents at least 10 experiments that were performed on 3 independent days. There was a significant difference in the FRAP recovery rates on the E6 gene under Pladienolide B treatment relative to the control (One way ANOVA, p<0.0001). Recovery curves with error bars appear in S2E Fig. (B) RNA FISH experiment to detect the distribution of the E6 mRNA in U2OS cells treated with Pladienolide B and overexpressing Clk1 (cyan) using a Cy5-labeled probe that detects the MS2 region of the E6 mRNA (yellow), and a Cy3-labeled probe that detects the intron of the E6 mini-gene (red). DIC in grey. Bar = 5 μm. (C) Semi-quantitative RT-PCR of cells treated with Pladienolide B (10 μM) or with Clk1 overexpression, together with dox induction (6 hrs). mRNA profiles were examined for the intron inclusion of DXO pre-mRNA and for the exon skipping of E6, MCL1, NOP56 and p27 pre-mRNAs. The positions of the different products are noted on the right. (D) Quantitative RNA FISH experiment that examined the ratio of intron/first exon of E3 and E6 pre-mRNAs in cells overexpressing Clk1 and in untreated cells. Each blue dot represents the ratio of intensities measured on a single transcription site. The experimental data (blue dots) were compared to the simulation output of the same experiment for short (50 sec, red dots) or long (11 min, green dots) transcript retention times. The splicing efficiency time was estimated in the simulation as depletion of the intron 750bp after it was created or 500bp when CLK1 was expressed.

Finally, we verified that Clk1 overexpression and dispersion of splicing factors in the nucleoplasm did not inhibit splicing, which could lead to the faster recovery of the E6 mRNA FRAP curves, as seen with splicing inhibitors spliceostatin A (SSA) [24] and Pladienolide B (PlaB) (Fig 6A). Clk1 overexpression did not lead to the nuclear accumulation of unspliced pre-mRNAs, as seen with RNA FISH probes that target the intronic sequences of the E6 transcript. Under splicing inhibition conditions, the intron sequences in the pre-mRNA were found throughout the nucleus and in nuclear speckles, whereas under Clk1 overexpression conditions the introns were only found at the site of transcription, as expected (Fig 6B). Also, the examination of splicing patterns of several genes as performed in [32] showed that Clk1 overexpression conditions differed from splicing inhibition conditions (Fig 6C). We therefore concluded that the nucleoplasmic dispersal and abundance of splicing factors by Clk1 overexpression does not inhibit splicing but can modulate the splicing rate.

In order to validate that the faster splicing was the reason for the increase in the release of the mRNA from the gene when Clk1 was overexpressed, we used another approach. The rate at which splicing takes place on a gene can be measured by quantitative RNA FISH. We have used the measurements of the relative ratio of introns to the exon on the active genes in our previous study [24] to extract information about the assumed kinetic rates of splicing on these specific genes.

When genes with increasing numbers of introns such as E3 and E6 were previously examined by quantitative RNA FISH, we found that the intron/exon ratio increased as expected when the number of introns in the gene was larger. These data were compared to a simulation of the data that gave the same expected results [24]. We concluded from this analysis that the outcome of the splicing process on the gene remained regular even for the E6 mRNA that was retained for longer times on the gene, although its transcription time had not changed. We found that when splicing was inhibited, the delay in the mRNA release was abolished, meaning that the mRNA transcript release from the gene was delayed until the splicing was fully completed. The intron/exon ratio kept its expected ratio even though the transcript was delayed, which also meant that the limiting factor was the completion of splicing that required extra time.

Considering the results obtained here showing that the abundance of splicing factors can modulate the rate of mRNA release from the gene, we returned to the simulation in order to check what are the expected values, given that the kinetics had changed. The simulation simulates the biological process in which sets of genes are transcribed simultaneously. Namely, the RNA Pol II can pause during elongation [33], and when transcription terminates there is a time delay until the transcript is released. Using this system, we simulated the FRAP experiments and retrieved kinetic parameters [24]. The only parameter that was changed between the fast and slow kinetics of the FRAP experiments was the transcript retention time on the gene due to splicing efficiency. We also used the simulation to predict the intron/exon ratio on the gene at steady state with the same kinetic parameters.

Now, we used the simulation again, this time calculating the expected intron/exon ratio when Clk1 was overexpressed, to test whether Clk1 overexpression accelerates or inhibits the splicing. When splicing occurs co-transcriptionally, the spliced intron will leave the gene shortly after transcription. The simulation predicts that the intron/exon ratio is only slightly reduced under Clk1 overexpression compared to untreated cells when the mRNA is simulated to be shortly retained on the gene (Fig 6D, red dots) but drops significantly if the retention time is increased (Fig 6D, green dots), since the spliced mRNA will delay on the gene after transcription is complete (as a result there will be accumulation of exons relative to the introns that were spliced and already left).

Then, we performed the quantitative RNA FISH experiment and examined the ratio of intron/exon of E3 and E6 pre-mRNAs in cells overexpressing Clk1. We found that this ratio slightly decreased when Clk1 was overexpressed compared to the control conditions, but the relative splicing efficiency between E3 and E6 did not change under the different conditions (Fig 6D, the experimental blue dots are similar to the simulated red dots). Although the FRAP experiment showed that E6 transcripts were delayed on the gene under regular conditions, the intron/exon ratio agreed with the simulated kinetic values when there was no RNA retention, and did not change with or without Clk1 overexpression, suggesting that splicing occurs faster with overexpression of Clk1. In other words, even though under Clk1 overexpression conditions there was less ‘time’ (the FRAP experiment on E6 pre-mRNA + Clk1) to perform the same splicing ‘work’ (the intron/exon ratio) compared to control conditions, the splicing output was the same, meaning that splicing occurs faster with overexpression of Clk1.

### Hyper-phosphorylation of splicing factors by Clk1 affects the release of the mRNA from the gene

**Fig 7:**
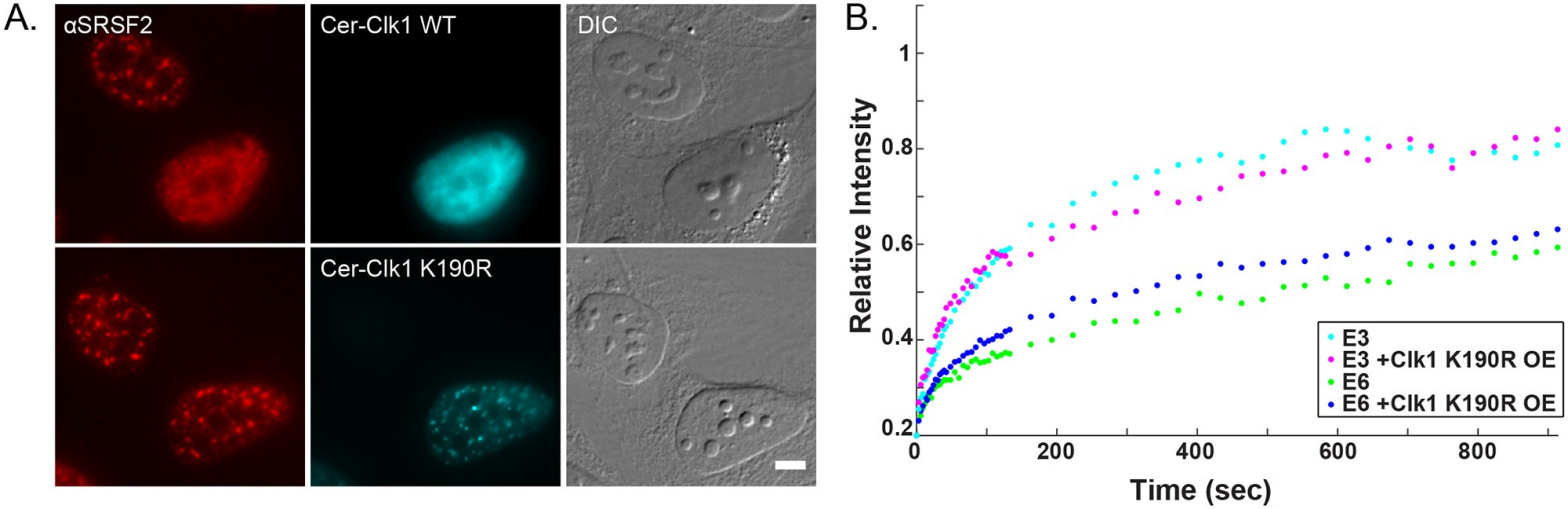
Overexpression of a catalytically inactive mutant of Clk1 (Clk1K190R) does not lead to splicing factor dispersal and does not influence the FRAP recovery rates on the E6 and E3 genes. (A) The distribution of SRSF2 (red, detected by immunofluorescence) in cells transfected with the normal or mutant Clk1 kinase tagged with Cerulean (cyan). DIC in grey. Bar = 5 μm. (B) Recovery curves of the YFP-MS2 mRNA FRAP measurements performed at the E3 and E6 transcription sites after the overexpression of mutant kinase Clk1. The relative intensity of each plot represents at least 10 experiments that were performed on 3 independent days. There were no significant differences in the FRAP recovery rates for the E6 and E3 genes after Clk1 K190R OE compared to control cells (One way ANOVA, p = 0.09268, p = 0.6710). Recovery curves with error bars appear in S2F Fig.

Next, we examined how Clk1 mediates this effect. Clk1 overexpression disperses the splicing factors in the nucleoplasm and causes their hyper-phosphorylation. We examined which of these effects was responsible for the faster release of the E6 mRNA from the gene. Overexpression of a mutant Clk1 (Clk1 K190R) lacking kinase activity did not disperse the splicing factors in the nucleoplasm [28, 29] and did not change the FRAP recovery curve of the E6 mRNA (Fig 7).

**Fig 8:**
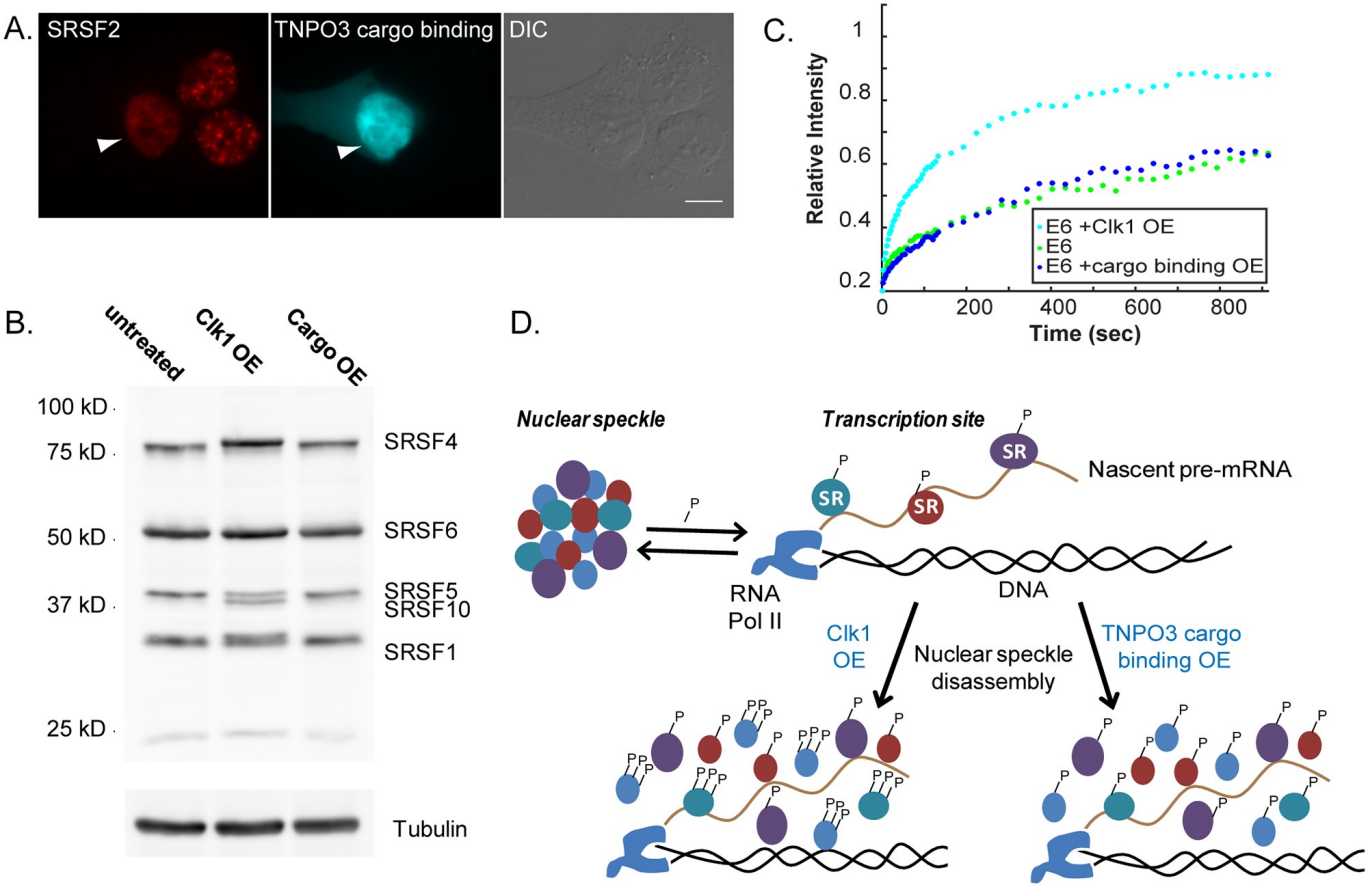
Nucleoplasmic dispersal of splicing factors without affecting the phosphorylation levels of the splicing factors does not alter the E6 FRAP recovery curves. (A) The distribution of SRSF2 (red, immunofluorescence) in cells transfected with the cargo binding domain of TNPO3 tagged with Cerulean (cyan). DIC in grey. Bar = 5 μm. (B) Western blot analysis with a MAb104 antibody that detects phosphorylated SR proteins performed on U2OS protein extracts from untreated cells and cells transfected with Clk1 or with the cargo binding domain of TNPO3. Tubulin was used as a loading control. (C) Recovery curves of the YFP-MS2 mRNA FRAP measurements performed on the E6 transcription sites in untreated cells and after transfection with Clk1 or the cargo binding domain of TNPO3. The relative intensity of each plot represents at least 10 experiments that were performed on 3 independent days. There were no significant differences in the FRAP recovery rates for the E6 gene after TNPO3 cargo binding domain overexpression compared to control cells (One way ANOVA, p = 0.7671). Recovery curves with error bars appear in S2G Fig. (D) Scheme describing the distribution and phosphorylation levels of splicing factors on the active gene in normal cells and in cells where splicing factors were dispersed in the nucleoplasm due to the overexpression of Clk1 or TNPO3 cargo binding domain.

Next, we wanted to disperse the splicing factors in the nucleoplasm without affecting their phosphorylation levels. We discovered that the overexpression of the cargo binding domain of TNPO3, a protein that is responsible for the nuclear import of splicing factors [34, 35], causes nuclear speckle disassembly (Figs 4B and 8A). This we found by reasoning that TNPO3 as a transporter protein contains a domain that is able to bind to its cargo, SR proteins. Hence, overexpression of the cargo binding domain only, might have a dominant negative effect and disrupt the interactions between the SR proteins, to prevent nuclear speckle formation. Indeed, we found that overexpression of the TNPO3 cargo binding domain dispersed the splicing factors in the nucleoplasm but did not change the phosphorylation pattern of the splicing factors compared to Clk1 overexpression (Fig 8B), did not prevent the splicing factors from being recruited to the active gene (S7A Fig) and did not cause a splicing defect as does Pladienolide B (S7B Fig). Finally, the overexpression of the TNPO3 cargo binding domain did not change the E6 mRNA FRAP curves (Fig 8C). This meant that in addition to the increased availability of splicing factors in the nucleoplasm affecting the release of the processed transcript from the gene, also the hyper-phosphorylation of the splicing factors is required.

## Discussion

Using live-cell imaging and quantitative analysis of splicing factor dynamics in the cell nucleus we could address whether the levels of splicing factors in the nucleoplasm can influence the rates at which a spliced mRNA is released from a gene. When we increased splicing factor abundance in the nucleoplasm, we found changes in splicing factor dynamics on active genes and an effect on the release of the mRNA from the gene (Fig 8D). We show that the dispersal of endogenous splicing factors in the nucleoplasm by Clk1 overexpression increased splicing factor dynamics at the active gene and in the nucleoplasm. Importantly, the more rapid interactions of splicing factors with the active gene coincided with a faster release of the retained mRNA from the gene, suggesting that splicing is completed faster. Under regular conditions this mRNA stalls on the gene probably due to many splicing events [24]. The connection between the splicing events occurring on the mRNA and the release time of the mRNA from the gene was previously proven by the inhibition of splicing, namely, rapid release from the gene was observed when splicing was inhibited using splicing inhibitors (PlaB or SSA). Now we show that the rapid release of the mRNA from the gene under conditions of Clk1 overexpression was not due to a splicing defect, rather it was dependent of splicing factor availability, resulting in more efficient splicing. This result is consistent with other studies showing that Clk1 can modulate the splicing of several genes [36–38].

Interestingly, we found that the overexpression of some splicing factors rather than the dispersal of all splicing factors in the nucleoplasm by Clk1 overexpression could also modify the release of mRNA from the gene. This was not observed with the overexpression of all splicing factors tested, and may indicate that some factors stay bound to the transcript after the splicing reaction or maybe affect a different set of genes, as some studies have shown [39, 40]. This might also relate to the changes in the expression of some splicing factors in cancer cells, which can contribute to the oncogenic pathways in these cells [41–43]. Moreover, despite the similarity between SR splicing factors, we showed that the deletion or replacement of the RS domain affects the localization and the activity of the splicing factors. The observation that only some of the splicing factors affect the release of the mRNA from the gene may explain why the depletion of Son and MALAT1 do not affect the gene expression rates. As reported, those two factors generate interactions with a specific set of nuclear speckle components [44–46] that do not definitely affect the transcription rate of the tested gene.

Since SR proteins undergo phosphorylation that regulates their activity during spliceosome assembly [47, 48], we assume that the dispersal of splicing factors in the nucleoplasm is not sufficient for changing the RNA release rate. It is known that Clk1 overexpression does not affect only the localization of splicing factors but also influences their phosphorylation levels [29]. For instance, it was demonstrated that during heat shock or osmotic stress, the SR proteins phosphorylation state is regulated by the maturation of Clk1 mRNAs, which cause an elevation in its protein levels [37]. Hence, it was interesting to understand whether increasing the levels of splicing factors in the nucleoplasm without affecting their phosphorylation levels would affect the mRNA release rates. We showed that dispersing splicing factors in the nucleoplasm without changing their phosphorylation levels, by overexpressing the cargo binding of TNPO3, did not affect the release rates. This mutant TNPO3 protein may disrupt an interaction between splicing factors to another molecule and hence prevent splicing factors from forming nuclear speckles but did not prevent their recruitment to the transcribing gene.

There is controversy regarding the possible roles of nuclear speckle structures. It was recently shown that nuclear speckles associate with specific genomic regions which may suggest that those structures can regulate gene expression [16, 17]. We suggest that nuclear speckles have a role in regulating or buffering the levels of splicing factors that are available for splicing in the nucleoplasm. How can such regulation transpire? It is possible that some splicing factors are limiting in the splicing reactions taking place in the nucleus. We have recently shown that different splicing factors can have different affinities for nuclear speckles and so the abundancies of the factors in the nucleoplasm may be unique to each factor [49]. On the other hand, several studies have shown that overexpression of splicing factors affects the splicing outcome, for example [50, 51]. Additionally, introns have different binding site sequences for splicing factors such that different factors are probably differentially recruited to pre-mRNAs. Altogether, we propose that if certain genes are more highly transcribed than others, then some pre-mRNAs will be spliced depending on the nucleoplasmic availability of the SR proteins that are required for their specific splicing.

Taken together, this study shows that gene expression rates can be tuned by modulating the levels of splicing factors that can participate in the splicing reaction. This mechanism is dependent not only on the localization of the splicing factors that are required at the gene, but also on the appropriate phosphorylation levels that enable the factors to interact with the nascent transcript and to assemble the spliceosome machinery.

## Materials and methods

### Plasmid construction

For plasmids encoding SRSF1-7, Clk1, SRPK1 or the TNPO3 cargo binding domain, the ORF of the gene was amplified by RT-PCR (GoTaq Green Mix, Biological Industries) from cDNA (RevertAid First Strand cDNA Synthesis Kit (Fermentas)) of U2OS cells, using primers that contain the appropriate restriction site as listed in S5 Table. The amplified products were subcloned into pCMV-HA, peRFP-C1 or pmCerulean-C1 (Clontech). The previously constructed Cerulean-Clk1 plasmid was mutated using the QuikChange II Site-Directed Mutagenesis kit (Stratagene). Lysine 190 was mutated to arginine using primers:

Forward- AGACGTGTAGCAGTAAGAATAGTTAAAAATGTG
Reverse-CACATTTTTAACTATTCTTACTGCTACACGTCT

Other plasmids have been previously described [24].

### Cell culture and transfections

Human U2OS E3 and E6 cells [24] were maintained in low glucose DMEM (Biological Industries, Beit-Haemek, Israel), supplemented with 10% fetal bovine serum (HyClone Laboratories, Logan, UT). For transient transfections, cells were transfected with 1–5 μg of plasmid DNA and 40 μg of sheared salmon sperm DNA (Sigma) when using electroporation (Gene Pulser Xcell, Bio-Rad).

For overexpression experiments, the PolyJet reagent (SignaGen) was used. Cells were induced to express the E3 and E6 genes by addition of 1 μg/ml doxycycline (Sigma). Transgenic U2OS E3 and E6 cell lines with stable integration of BACs carrying C-terminally tagged SC35 (SRSF2), SRp75 (SRSF4), SRp40 (SRSF5), SRp55 (SRSF6), 9G8 (SRSF7), U1-70K, U2AF65, and PRP8, were generated as described [26]. For splicing inhibition, cells were treated for 6 hrs with Pladienolide B (10 μM, Santa Cruz).

### Total RNA purification

Total RNA was isolated using Tri-Reagent (Sigma). DNA-free Kit (Ambion) was used to remove genomic DNA contamination. cDNA (1 μg RNA) was synthesized using the ReverseAid First Strand cDNA Synthesis Kit (Fermentas) with oligo-dT as a primer. Semi-quantitative RT-PCR was performed using an Eppendorf Thermocycler amplification for 19–38 cycles (depending on the saturation level of the genes amplified) using 1 min denaturation at 94°C, 1 min annealing at 55°C, 1 min extension at 72°C; and 72°C for 10 min for final extension. Primers for:

E6 forward: TCTGACACAACTGTGTTCAC
E6 reverse: TCCACGTGCAGCTTGTCACA
p27 forward: CAGCTTGCCCGAGTTCTACT
p27 reverse: GTCCATTCCATGAAGTCAGCG
DXO forward: TGGGGAGGTTAACACCAACG
DXO reverse: GCTCTGGGAAAGCTA AGGA
MCL1 forward: GAGGAGGAGGAGGACGAGTT
MCL1 reverse: ACCAGCTCCTACTCCAGCAA
NOP56 forward: GCATCCACAGTGCAGATCCT
NOP56 reverse: GCAATCGATTCGTGAGGCAA
Clk1 Forward: GCATAGTAGCAAGTCCTCTG
Clk1 Reverse: TACTGCTACACGTCTACCTC

### siRNA knockdowns

Cells were transfected with siRNA for Son (Cat#D-012983-01, Thermo Scientific) or a negative scrambled control (Cat#D-001210-05), using Lipofectamine 2000. mRNA expression levels of Son were examined by real time qRT-PCR (performed 3 times for scrambled and for each treatment) from total RNA using Tri Reagent (Sigma) 72 hrs after siRNA transfection, and normalized to β-actin mRNA levels. Primers used: Son forward- 5’ GTGGAACCAAGCCACAAAGT. Son reverse- 5’ CCTTGGACTACCTTCCCACA. ActB forward– 5’ GCACAGAGCCTCGCCTT. ActB reverse- 5’ CCTTGCACATGCCGGAG.

### MALAT1 knock down using CRISPR-Cas9

Two single-guide RNAs (sgRNAs) targeting the TATA box and first exon of human *MALAT1* gene were designed: sgRNA1- TACGCCTCGCCCGAGCTGTG, sgRNA2- AGGTTTCTAAAAACATGACGG. The sgRNAs were cloned into a Cas9-2A-GFP plasmid (Addgene #48138) according to [52] and transfected into U2OS cells using the PolyJet reagent. Three days after transfection, the GFP positive cells were sorted into a 96 plate using FACSAriaIII, and positive colonies were screened. PCR was performed on genomic DNA (TIANGAN #DP304-02) using primers: Forward- AAGCAGTTGGGGGAGAAAGT. Reverse- GCGTCATGGATTTCAAGGTC. The primers that were used for the end of the gene: Forward- GGCAGGAGAGACAACAAAGC. Reverse- AGCACCTGCAGAGAAAAGGA.

### Western blotting

Cells were washed in cold PBS, and proteins were extracted in RIPA lysis buffer (50 mM Tris pH 8.0, 5 mM EDTA, 150 mM NaCl, 0.5% Nonidet P-40) containing 10 mM Na-flouride, 1 mM Na-orthovanadate, protease inhibitor cocktail (Sigma), 2.8 μg/ml aprotinin and 1 mM PMSF, and placed on ice for 20–25 min. The resulting lysate was centrifuged at 10,000 rpm for 10 min at 4°C. 20–40 μg/μl of protein/lane was run on SDS-polyacrylamide gels and transferred to a nitrocellulose membrane (0.45 μm). The membrane was blocked in 5% BSA, and then probed with a primary antibody for 2 hrs at room temperature (RT), followed by incubation with a HRP-conjugated goat anti-rabbit/mouse IgG (Sigma) for 1 hr at RT. For loading control, the blots were reblotted with an anti-α-tubulin antibody (Abcam). Immunoreactive bands were detected by the Enhanced Chemiluminescence kit (ECL, Pierce). Primary antibodies used were mouse anti-MAb104 that was purified from hybridoma cells (MAb104 (ATCC CRL-2067)).

### Immunofluorescence

Cells were grown on coverslips, washed with PBS and fixed for 20 min in 4% paraformaldehyde (PFA). Cells were then permeabilized in 0.5% Triton X-100 for 3 min. After blocking, cells were immunostained for 1 hr with a primary antibody, and after subsequent washes the cells were incubated for 1 hr with secondary fluorescent antibodies. Primary antibodies: anti-SRSF2 (Sigma). Secondary antibodies: Alexa488-labeled goat anti-mouse IgG (Abcam) and Alexa594-labeled goat anti-mouse. Nuclei were counterstained with Hoechst 33342 and coverslips were mounted in mounting medium.

### Fluorescence *in situ* hybridization

Cells were grown on coverslips and fixed for 20 min in 4% PFA, and overnight with 70% ethanol at 4°C. The next day cells were washed with 1x PBS and treated for 2.5 min with 0.5% Triton X-100. Cells were washed with 1x PBS and incubated for 10 min in 40% formamide (4% SSC). Cells were hybridized overnight at 37°C in 40% formamide with a specific fluorescently-labeled Cy3 DNA probe (~10 ng probe, 50 mer). The next day, cells were washed twice with 40% formamide for 15 min and then washed for two hours with 1X PBS. Nuclei were counterstained with Hoechst 33342 and coverslips were mounted in mounting medium. The probe for the MS2 binding site was:

CTAGGCAATTAGGTACCTTAGGATCTAATGAACCCGGGAATACTGCAGAC. The intron probe was from [24]. In some cases, immunofluorescence was performed after the RNA FISH using the standard protocol.

FISH experiments with Stellaris (Biosearch Technologies) probes for MALAT1 were performed according to the manufacturer’s adherent cell protocol. To reduce photobleaching, the cells were submerged in GLOX buffer (pH = 8, 10 mM, 2x SSC, 0.4% glucose), supplemented with 3.7 ng of glucose oxidase (Sigma G2133-10KU) and 1 μl Catalase (Sigma 3515), prior to imaging [53, 54].

### Quantitative RNA FISH

Quantitative RNA FISH was performed as previously described [24, 55], in which the ratio of exon and intron FISH signals from two different probes in two different channels is measured and compared. U2OS cells expressing the E6 mRNA were treated under different conditions, and then hybridized with RNA FISH probes to the first exon (Cy5) and the intron (Cy3). Z stacks (200 nm steps) of the RNA FISH samples were collected. All cells were imaged on the same day and under identical conditions. Images were deconvolved using Huygens (SVI, The Netherlands). The sum of the pixel values of the transcription sites in each channel was measured using Imaris (Bitplane, MN), and the ratio between the channels was calculated.

### Fluorescence microscopy, live-cell imaging and data analysis

Wide-field fluorescence images were obtained using the Cell^R system based on an Olympus IX81 fully motorized inverted microscope (60X PlanApo objective, 1.42 NA) fitted with an Orca-AG CCD camera (Hamamatsu) driven by the Cell^R software. Live-cell imaging was carried out using the Cell^R system with rapid wavelength switching. For time-lapse imaging, cells were plated on glass-bottomed tissue culture plates (MatTek, Ashland, MA) in medium containing 10% FBS at 37°C. The microscope is equipped with an incubator that includes temperature and CO_2_ control (Life Imaging Services, Reinach, Switzerland).

For counting of nuclear speckles, SRSF7-GFP cells were untransfected or transfected with either RFP-CLK1 or RFP-TNPO3. 24 hrs post-transfection, cells were fixed and imaged under the same conditions. Nuclear speckles detection was performed using the ImageJ "find maxima" function, with noise tolerance value set to 200. For measuring the nuclear to cytoplasmic (n/c) ratio of SRSF4, the average fluorescence intensity of GFP-SRSF4 or GFP-SRSF4 with no RS domain was measured in a representative area in the nucleus and cytoplasm of the cells using the ROI measurement function in Xcellence software.

### Fluorescence recovery after photobleaching (FRAP)

E3 cells were maintained in Phenol Red–free Leibovitz’s L-15 with 10% FCS at 37°C. In order to mark active transcription sites, cells were transfected with MS2-CP-mCherry using PolyJet (SignaGen Laboratories), and then transcriptionally induced with doxycycline (1 μg/mL) for several hours. For Clk1 overexpression, cells were co-transfected with HA-Clk1 and MS2-CP-mCherry. FRAP experiments were performed 1 day later. FRAP image sequences were obtained on an Olympus FV1000 inverted scanning confocal microscope with a heated chamber and objective heater (37°C) and a 60×, 1.35 NA oil objective. Cells were scanned using a 488 nm laser for detection of GFP-tagged U1-70K, SRSF6, SRSF2, SRSF7, SRSF4, SRSF5, U2AF65 or PRP8 and a 561 nm laser for the detection of MS2-CP-mCherry. GFP-tagged splicing factors were bleached using the 488 nm laser in the nucleoplasm, speckles, and at the active transcription site. Five pre-bleach images were acquired. Post-bleach images were acquired at a frequency of four images per 2 sec. For analysis of fluorescence recovery, FRAP data were normalized and calculated, as previously described [56].

FRAP experiments on mRNA were performed using a 3D-FRAP system (Photometrics) built on an Olympus IX81 microscope (636 Plan- Apo, 1.4 NA) equipped with an EM-CCD (Quant-EM, Roper), 491 nm lasers, Lambda DG-4 light source (Sutter), XY&Z stages (Prior), and driven by MetaMorph (Molecular Devices). Experiments were performed at 37°C with 5% CO_2_ using a live-cell chamber system (Tokai). For each acquisition at least 7 z-slices were taken every 350 nm. For FRAP, six pre-bleach images were acquired. Post-bleach images were acquired in three time intervals. The first interval was taken for 45 sec every 3 msec, the second interval was taken for 90 sec every 6 sec and the last interval was taken for 480 sec every 30 msec. FRAP experiments were analyzed using lab-written ImageJ macros previously described [57].

### Computational simulation

Monte Carlo simulations (Matlab) of the transcriptional process were based on the mechanistic models as described in [24]. Briefly, the FRAP experiments were simulated, and by fitting the simulation to the experimental data the kinetic parameters of the process were retrieved. The simulation performs stochastic decisions by using random numbers obtained from the simulation and checks whether they are smaller than the kinetic parameters. For each gene (E3 and E6), a set of identical arrays was generated. When a polymerase moves through the ‘‘MS2 region” of the gene, the mRNA accumulates ‘‘fluorescence” that is maintained until the end of the gene. Polymerases could randomly enter and exit a paused state during elongation [33], and were stochastically released at a given termination rate [58]. The simulation reached steady state after long times, as expected. We used the same kinetic parameters calculated previously [24], where the difference between the fast (E3, and E6 with CLK1) to the slow kinetics (E6) was only the retention time (50 sec and 11 min, respectively). In order to obtain the kinetic parameters for steady state, the RNA FISH intron/exon ratio was calculated by the simulation (S6 Table), where the ‘splicing efficiency’ means the time after intron creation.

### Statistical analysis

Linear mixed-effects modeling were used to test the effect of different treatments on the relative intensity of the fluorescence recovery as a function of time. All values were log2-transformed to meet normality assumption except the data shown in Fig 2. Specifically, second-degree polynomial linear mixed-model regressions were fitted, with time as the continuous predictor and treatment as a fixed effect. Experiments were defined as a random effect. Post-hoc analysis was performed in terms of linear contrasts between treatments and p-values were corrected for multiple testing using the FDR procedure.

## Supporting information

S1 Fig Measuring the delay in the release of the E6 transcripts from the active gene following transcription. (A) The E6 mRNA (detected by RNA FISH with an MS2 probe, red) co-localized with the YFP-MS2 protein (green) that detects the transcript. Merge in yellow and DIC in grey. Bar = 5 μm. (B) Recovery curves of the YFP-MS2 mRNA FRAP measurements performed on the E3 and E6 transcription sites. The relative intensity of each plot represents at least 10 experiments that were performed on 3 independent days. There was a significant difference in the FRAP recovery rates between the E6 and E3 genes (One way ANOVA, p<0.0001).
(TIF)

S2 Fig FRAP curves with error bars from (A) Fig 3A, (B) Fig 3B, (C) Fig 3C, (D) Fig 5B, (E) Fig 6A, (F) Fig 7B, (G) Fig 8C. (TIF)

S3 Fig SRSF4 lacking the RS domain still localizes in the nucleus. (A) The distribution of SRSF4 and SRSF4-no RS (green) in cells transfected with SRSF4-GFP and SRSF4 no RS-GFP. Hoechst nuclear staining in blue. DIC in grey. Bar = 10 μm. (B) The signals in the cytoplasm and the nucleus were measured and nucleus/cytoplasm (n/c) ratios were calculated. n = 47 cells, SRSF4; 39 cells, SRSF4 no RS. ***p<0.001.
(TIF)

S4 Fig Son depletion does not affect the recruitment of splicing factors to the active gene and does not influence the FRAP recovery rates of the E6 gene. (A) Nuclear speckle integrity was detected using SRSF7-GFP under Son depletion conditions. Hoechst DNA stain is in blue. Boxed regions in the images are shown in enlarged boxes. Bar = 5 μm. (B) Real time qRT-PCR analysis of Son mRNA levels in control and cell transfected with siRNA for 72 hrs. Data were normalized by the level of β-actin mRNA levels. The average quantification of 3 repeated experiments is presented in the plots (mean ± sd). A two-tailed *t* test was performed. **P < 0.01. (C) Recovery curves of the YFP-MS2 mRNA FRAP measurements performed on the E3 and E6 transcription sites after Son depletion. The relative intensity of each plot represents at least 10 experiments that were performed on 3 independent days. There were no significant differences in the FRAP recovery rates for the E6 and E3 genes under Son depletion conditions relative to the control (One way ANOVA, p = 0.0581, p = 0.067). (D) SRSF7-GFP (green) is recruited to the locus of E6 gene (detected by RFP-LacI) in Son depleted U2OS cells.
(TIF)

S5 Fig MALAT1 depletion does not affect the FRAP recovery rates on the E6 gene. (A) MALAT1 mRNA (detected by RNA FISH, red) is not enriched at the transcription site of the E6 active gene (RNA FISH with a probe to the CFP region in the E6 mRNA) under normal conditions and after Clk1 overexpression (cyan). Bar = 5 μm. (B) Depletion of MALAT1 (red) does not affect the transcriptional activity or the subcellular localization of the E6 mRNA (RNA FISH) in MALAT1 knockout cells. DIC in grey. Bar = 5 μm. (C) MALAT1 knockout was performed using two sgRNAs and was validated by PCR on genomic DNA from E6 U2OS cells using primers that span the deletion region and primers from the end of the gene (positive control). (D) MALAT1 depletion does not affect the recovery curves of the YFP-MS2 mRNA FRAP measurements performed on E6 active transcription sites. The relative intensity of each plot represents at least 10 experiments that were performed on 3 independent days. There was no significant difference in the FRAP recovery rates between the E6 gene with and without MALAT1 KO (One way ANOVA, p = 0.6792).
(TIF)

S6 Fig MALAT1 does not affect the recruitment of splicing factors to the active gene. The recruitment of the GFP tagged splicing factors SRSF1, SRSF2, SRSF3, SRSF6 and SRSF7 (green) to the transcription site of the E6 active gene (RNA FISH with a probe to MS2, red) was examined under normal conditions and after depletion of MALAT1 (RNA FISH, magenta). Cytoplasmic dots are CFP-peroxisomes seen in the GFP channel. DIC in grey. Bar = 5 μm.
(TIF)

S7 Fig TNPO3 expression does not cause a splicing defect. (A) RNA FISH experiment shows that the SRSF7 splicing factor (green) is recruited to the active E3 gene (probe to the MS2 region, magenta) when TNPO3 is overexpressed (cyan). Arrows point to the active transcription sites. (B) RNA FISH experiment to detect the distribution of the E6 mRNA in U2OS cells treated with Pladienolide B and overexpressing TNPO3 (cyan) using a Cy5-labeled probe that detects the MS2 region of the E6 mRNA (yellow), and a Cy3-labeled probe that detects the intron of the E6 mini-gene (red). DIC in grey. Bar = 5 μm.
(TIF)

S1 Movie Live-cell imaging of nuclear speckles disassembly. SRSF7-GFP cells were transfected with RFP-CLK1. 3 hrs post-transfection the cells were imaged every 10 minutes. Left, RFP-CLK1 signal (inverted, pseudocolored black). Right, SRSF7-GFP signal (green). Number of nuclear speckles decreased along with the increase in RFP-CLK1 expression. Time in minutes is shown in the bottom-right corner.
(AVI)

S1 Table The statistical differences between FRAP experiments that were performed on several splicing factors in three different sub-nuclear compartments: On the active gene, in the nucleoplasm, and in nuclear speckles (Fig 2). (DOCX)

S2 Table The exponential equations used to fit the FRAP curves and the relative fractions of the bi-exponential fits (Fig 2). (DOCX)

S3 Table The statistical differences between FRAP experiments which were performed on the E6 gene in cells overexpressing several splicing factors compared to control cells (Fig 3). (DOCX)

S4 Table The statistical differences between FRAP experiments that were performed on the splicing factors SRSF7, PRP8, SRSF2 and U1-70K on the active gene and in the nucleoplasm in cells overexpressing Clk1 relative to control cells (Fig 4). (DOCX)

S5 Table The list of primers containing the appropriate restriction sites used to amplify the ORF of several genes to create the plasmids encoding SRSF1-7, Clk1, SRPK1 and the TNPO3 cargo binding domain. (DOCX)

S6 Table Simulation results for intron/exon ratio calculations during steady states for the E3 and E6 genes with the two different retention times. Calculated for different splicing efficiency values. Normalized by E3 control data.
(DOCX)

S1 Data Excel files containing the data used to generate the plots presented in the figures of this study. (RAR)
