# Supplementary figures and images for: Availability of splicing factors in the nucleoplasm can regulate the release of mRNA from the gene after transcription
Source: PLoS Genet. 2019 Nov 25;15(11):e1008459. doi: 10.1371/journal.pgen.1008459 (PMC6901260; doi:10.1371/journal.pgen.1008459)

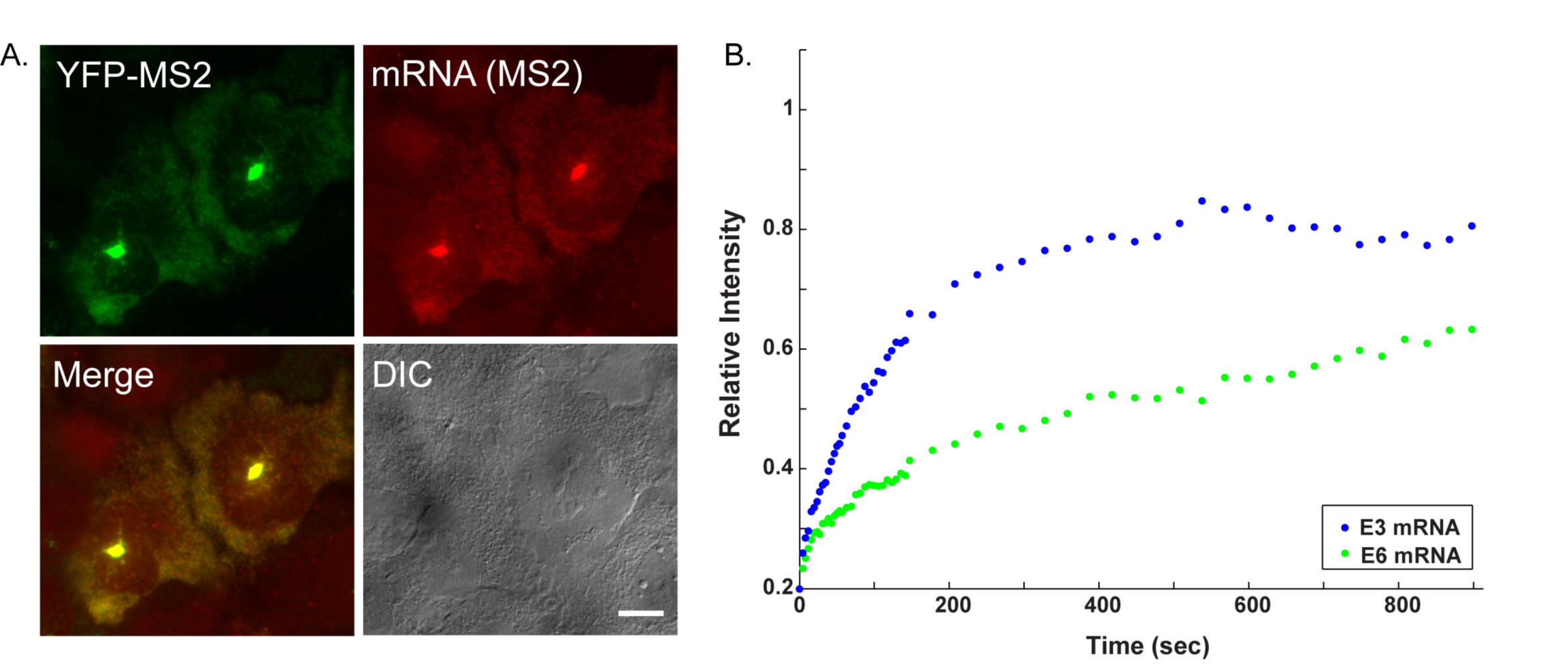

Supplement: S1 Fig — (A) The E6 mRNA (detected by RNA FISH with an MS2 probe, red) co-localized with the YFP-MS2 protein (green) that detects the transcript. Merge in yellow and DIC in grey. Bar = 5 μm. (B) Recovery curves of the YFP-MS2 mRNA FRAP measurements performed on the E3 and E6 transcription sites. The relative intensity of each plot represents at least 10 experiments that were performed on 3 independent days. There was a significant difference in the FRAP recovery rates between the E6 and E3 genes (One way ANOVA, p<0.0001). (TIF) [file pgen.1008459.s001.tif]

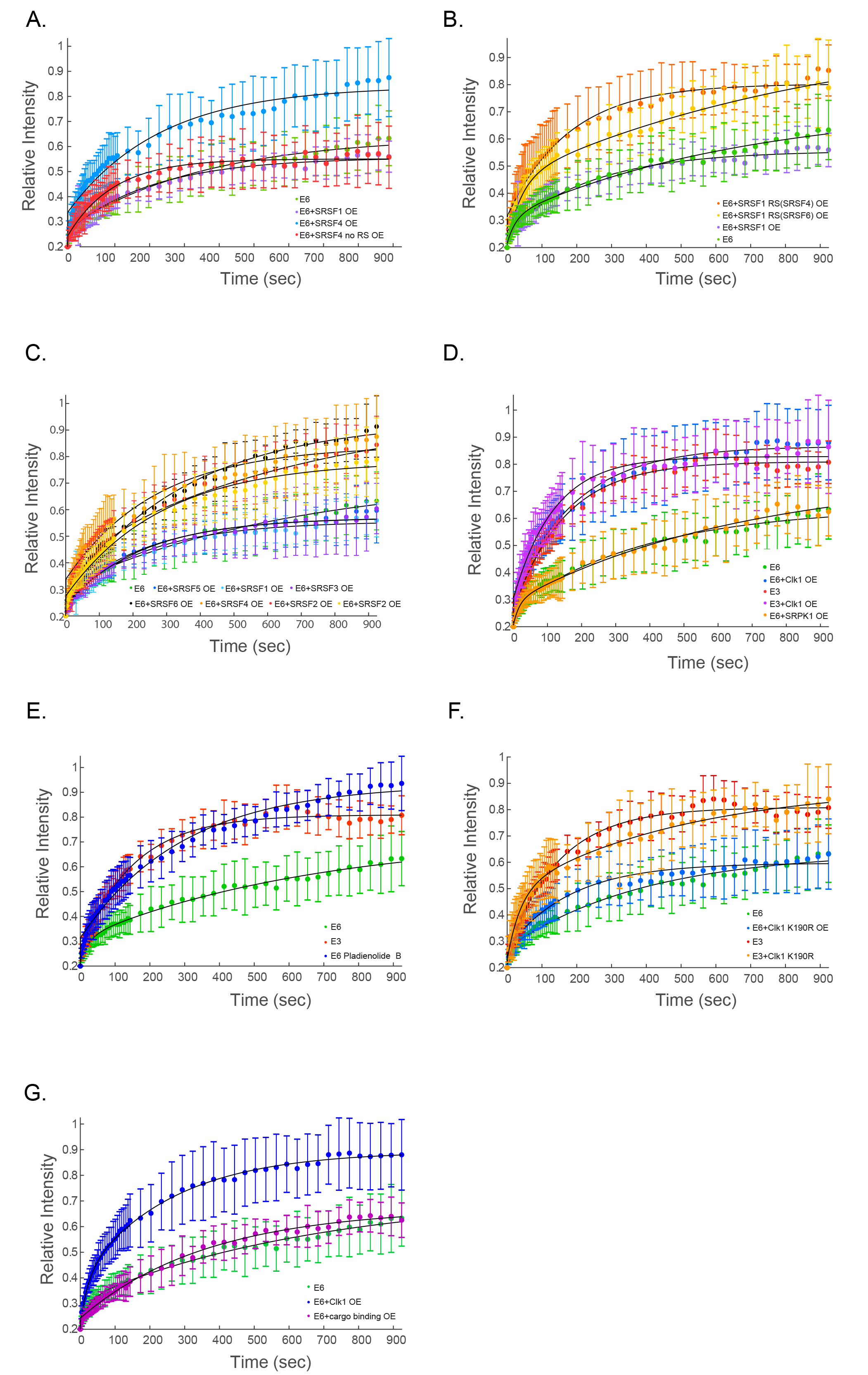

Supplement: S2 Fig — (TIF) [file pgen.1008459.s002.tif]

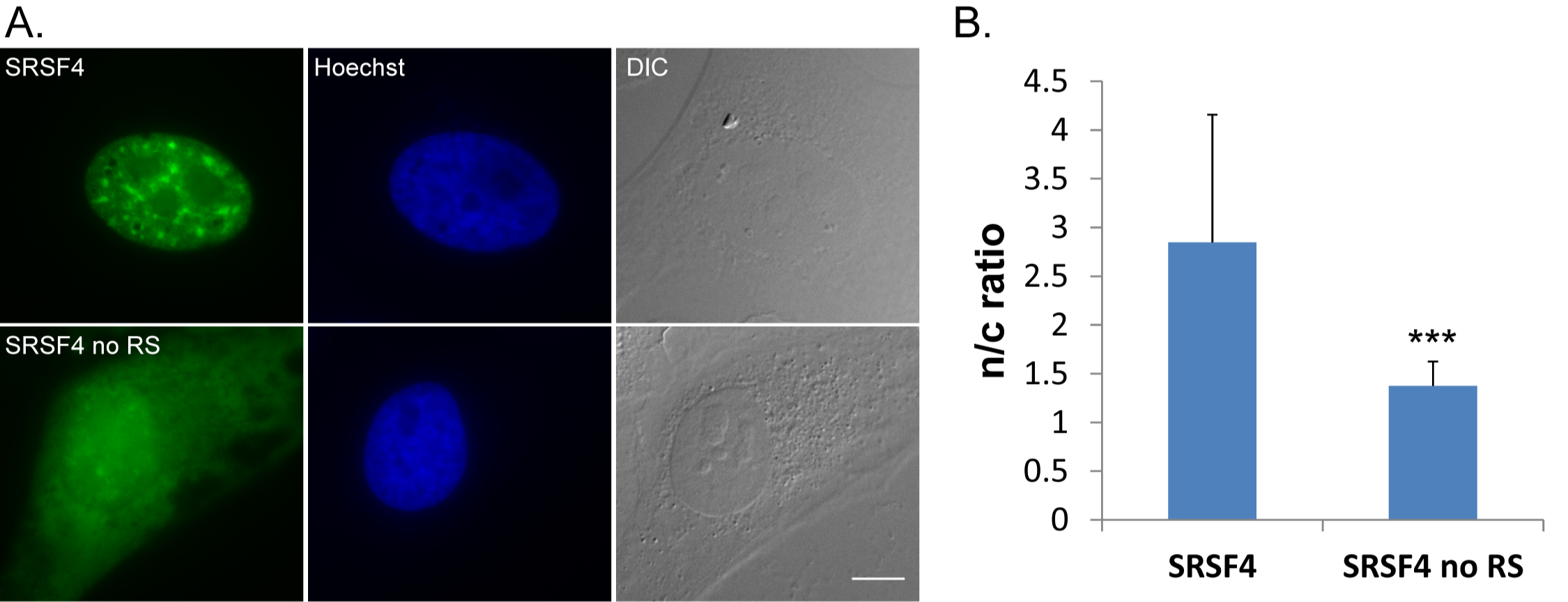

Supplement: S3 Fig — (A) The distribution of SRSF4 and SRSF4-no RS (green) in cells transfected with SRSF4-GFP and SRSF4 no RS-GFP. Hoechst nuclear staining in blue. DIC in grey. Bar = 10 μm. (B) The signals in the cytoplasm and the nucleus were measured and nucleus/cytoplasm (n/c) ratios were calculated. n = 47 cells, SRSF4; 39 cells, SRSF4 no RS. ***p<0.001. (TIF) [file pgen.1008459.s003.tif]

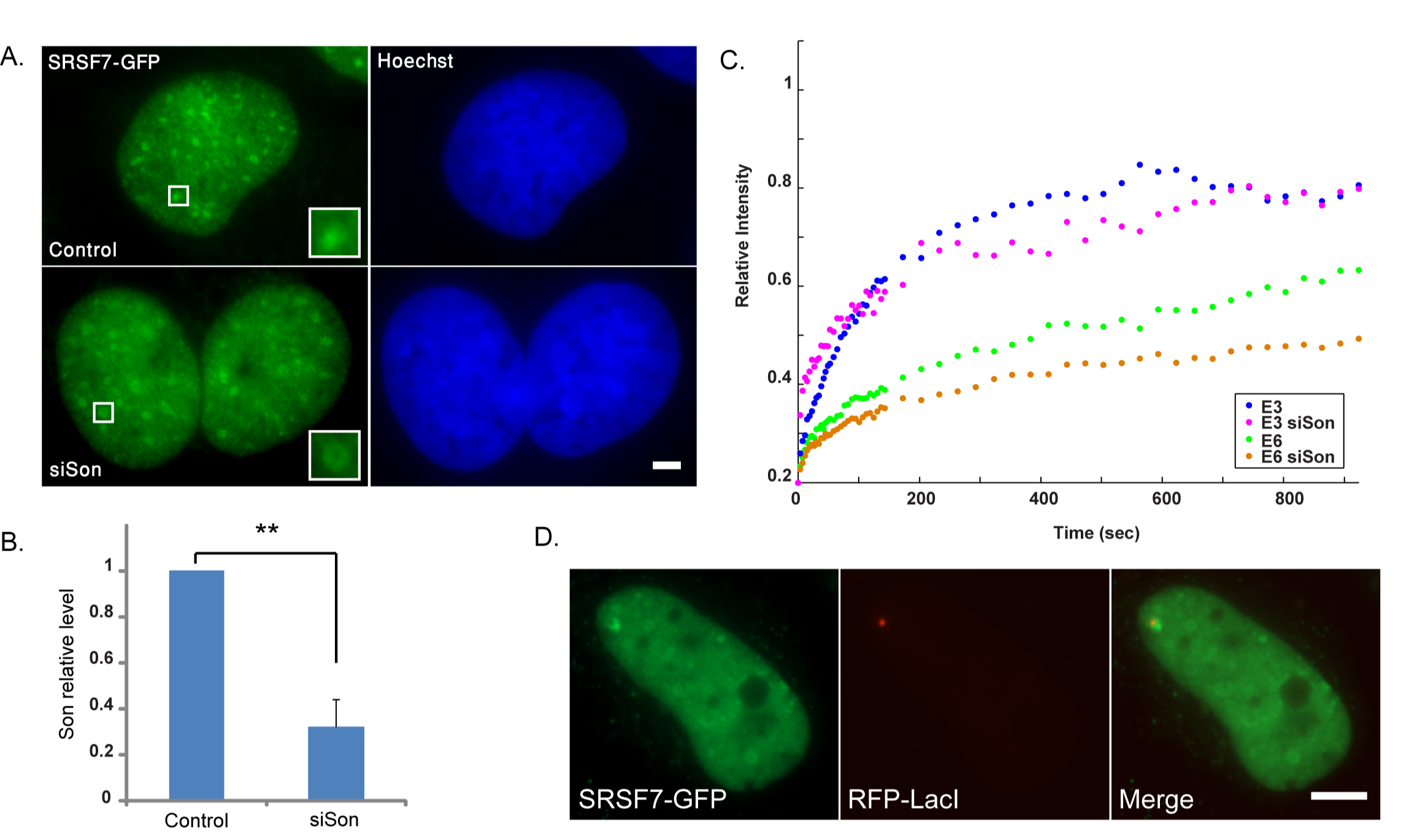

Supplement: S4 Fig — (A) Nuclear speckle integrity was detected using SRSF7-GFP under Son depletion conditions. Hoechst DNA stain is in blue. Boxed regions in the images are shown in enlarged boxes. Bar = 5 μm. (B) Real time qRT-PCR analysis of Son mRNA levels in control and cell transfected with siRNA for 72 hrs. Data were normalized by the level of β-actin mRNA levels. The average quantification of 3 repeated experiments is presented in the plots (mean ± sd). A two-tailed t test was performed. **P < 0.01. (C) Recovery curves of the YFP-MS2 mRNA FRAP measurements performed on the E3 and E6 transcription sites after Son depletion. The relative intensity of each plot represents at least 10 experiments that were performed on 3 independent days. There were no significant differences in the FRAP recovery rates for the E6 and E3 genes under Son depletion conditions relative to the control (One way ANOVA, p = 0.0581, p = 0.067). (D) SRSF7-GFP (green) is recruited to the locus of E6 gene (detected by RFP-LacI) in Son depleted U2OS cells. (TIF) [file pgen.1008459.s004.tif]

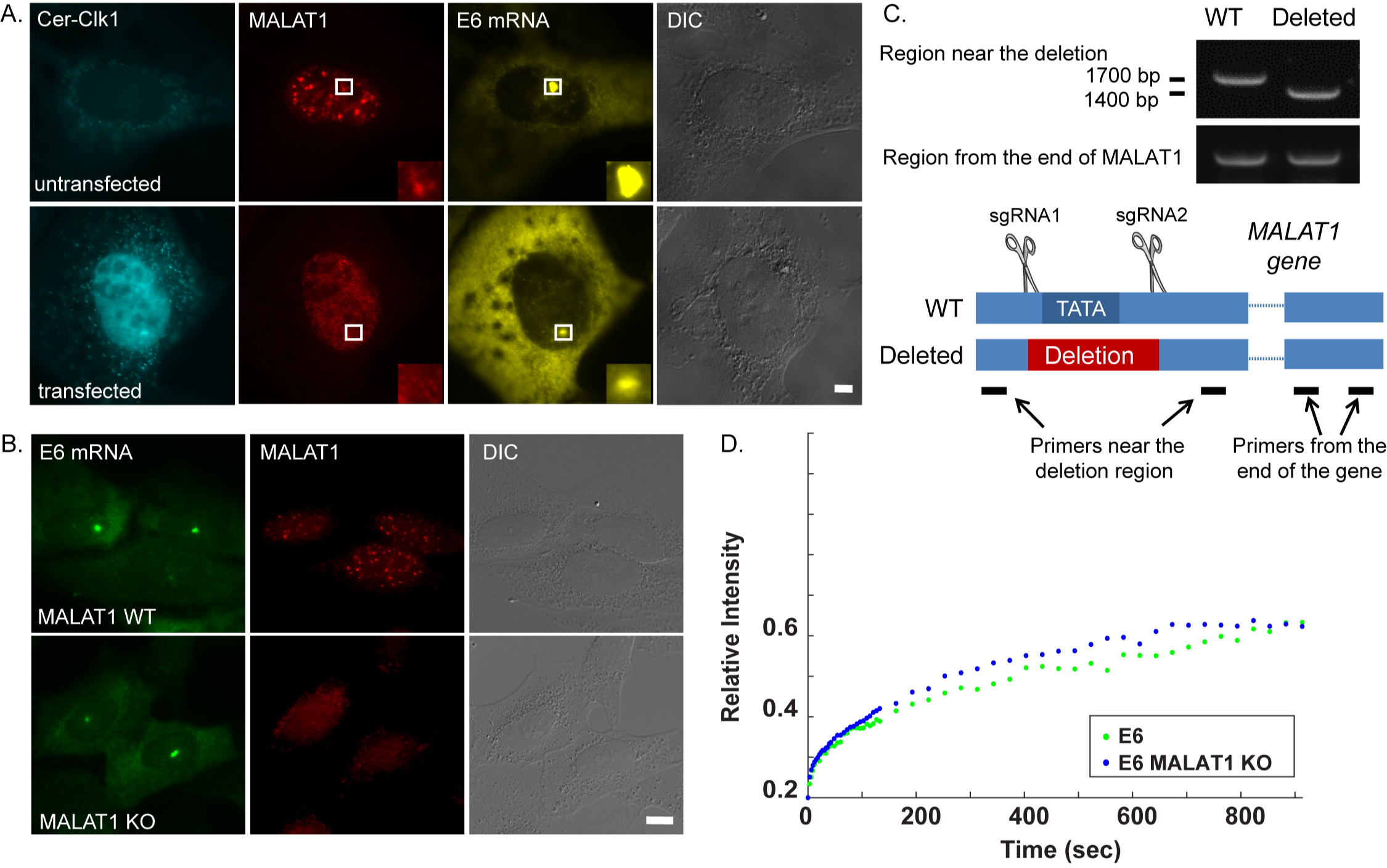

Supplement: S5 Fig — (A) MALAT1 mRNA (detected by RNA FISH, red) is not enriched at the transcription site of the E6 active gene (RNA FISH with a probe to the CFP region in the E6 mRNA) under normal conditions and after Clk1 overexpression (cyan). Bar = 5 μm. (B) Depletion of MALAT1 (red) does not affect the transcriptional activity or the subcellular localization of the E6 mRNA (RNA FISH) in MALAT1 knockout cells. DIC in grey. Bar = 5 μm. (C) MALAT1 knockout was performed using two sgRNAs and was validated by PCR on genomic DNA from E6 U2OS cells using primers that span the deletion region and primers from the end of the gene (positive control). (D) MALAT1 depletion does not affect the recovery curves of the YFP-MS2 mRNA FRAP measurements performed on E6 active transcription sites. The relative intensity of each plot represents at least 10 experiments that were performed on 3 independent days. There was no significant difference in the FRAP recovery rates between the E6 gene with and without MALAT1 KO (One way ANOVA, p = 0.6792). (TIF) [file pgen.1008459.s005.tif]

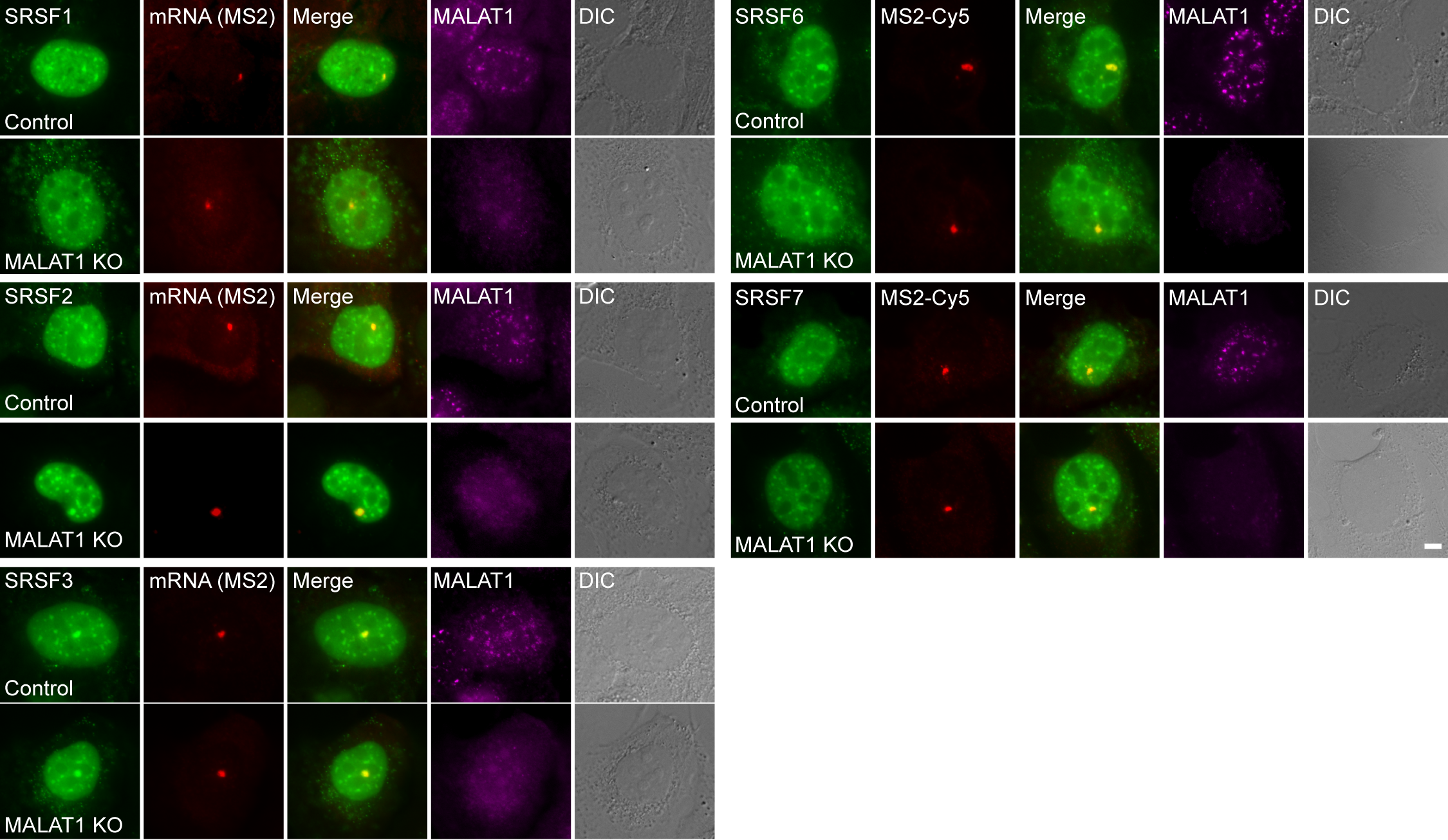

Supplement: S6 Fig — The recruitment of the GFP tagged splicing factors SRSF1, SRSF2, SRSF3, SRSF6 and SRSF7 (green) to the transcription site of the E6 active gene (RNA FISH with a probe to MS2, red) was examined under normal conditions and after depletion of MALAT1 (RNA FISH, magenta). Cytoplasmic dots are CFP-peroxisomes seen in the GFP channel. DIC in grey. Bar = 5 μm. (TIF) [file pgen.1008459.s006.tif]

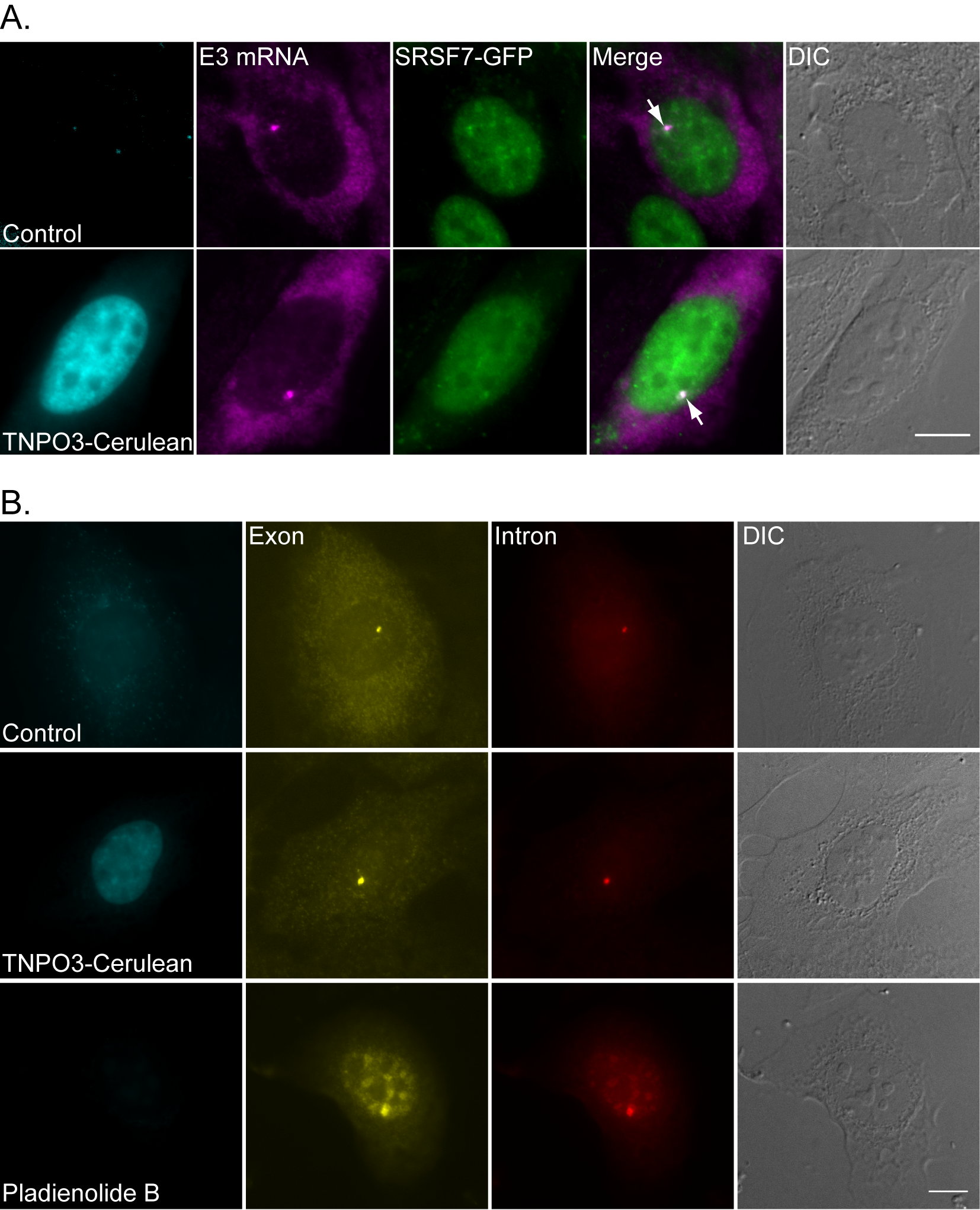

Supplement: S7 Fig — (A) RNA FISH experiment shows that the SRSF7 splicing factor (green) is recruited to the active E3 gene (probe to the MS2 region, magenta) when TNPO3 is overexpressed (cyan). Arrows point to the active transcription sites. (B) RNA FISH experiment to detect the distribution of the E6 mRNA in U2OS cells treated with Pladienolide B and overexpressing TNPO3 (cyan) using a Cy5-labeled probe that detects the MS2 region of the E6 mRNA (yellow), and a Cy3-labeled probe that detects the intron of the E6 mini-gene (red). DIC in grey. Bar = 5 μm. (TIF) [file pgen.1008459.s007.tif]
