# Supplementary material for: Availability of splicing factors in the nucleoplasm can regulate the release of mRNA from the gene after transcription
Source: PLoS Genet. 2019 Nov 25;15(11):e1008459. doi: 10.1371/journal.pgen.1008459 (PMC6901260; doi:10.1371/journal.pgen.1008459)
Supplement: S2 Table — (DOCX) [file pgen.1008459.s010.docx]

| **Fast fraction** | **Slow fraction** | **Fit equation** | **SF** |
| --- | --- | --- | --- |
| 0.8515 | 0.1485 | $f(t)=-0.14*exp(-0.0836*t) + -0.593*exp(-1.04*t) +0.943$ | SC35 Nucleoplasm |
| 0.7508 | 0.2492 | $f(t)=-0.233*exp(-0.212*t) + -0.553*exp(-1.25*t) +0.935$ | SC35 Speckles |
| 0.7798 | 0.2202 | $f(t)=-0.205*exp(-0.13*t) + -0.591*exp(-1.01*t) +0.931$ | SC35 Transcription site |
| 0.6748 | 0.3252 | $f(t)=-0.305*exp(-0.115*t) + -0.55*exp(-0.764*t) +0.938$ | SRSF4 Nucleoplasm |
| 0.4912 | 0.5088 | $f(t)=-0.434*exp(-0.152*t) + -0.377*exp(-0.805*t) +0.853$ | SRSF4 Speckles |
| 0.6157 | 0.3843 | $f(t)=-0.352*exp(-0.0921*t) + -0.477*exp(-0.574*t) +0.916$ | SRSF4 Transcription site |
| 0.3716 | 0.6284 | $f(t)=-0.301*exp(-0.0244*t) + -0.122*exp(-0.714*t) +0.479$ | Prp8 Nucleoplasm |
| 0.2065 | 0.7935 | $f(t)=-0.292*exp(-0.0217*t) + -0.055*exp(-89.3*t) +0.368$ | Prp8 Speckles |
| 0.2842 | 0.7158 | $f(t)=-0.262*exp(-0.0204*t) + -0.0697*exp(-0.593*t) +0.366$ | Prp8 Transcription site |
| 0.7552 | 0.2448 | $f(t)=-0.223*exp(-0.168*t) + -0.55*exp(-1.13*t) +0.911$ | SRSF6- Nucleoplasm |
| 0.6685 | 0.3315 | $f(t)=-0.305*exp(-0.133*t) + -0.52*exp(-0.79*t) +0.92$ | SRSF6 Speckles |
| 0.5718 | 0.4282 | $f(t)=-0.379*exp(-0.185*t) + -0.405*exp(-0.985*t) +0.885$ | SRSF6 Transcription site |
| 0.7913 | 0.2087 | $f(t)=-0.187*exp(-0.099*t) + -0.514*exp(-0.989*t) +0.896$ | SRSF5 Nucleoplasm |
| 0.6389 | 0.3611 | $f(t)=-0.325*exp(-0.228*t) + -0.455*exp(-1.46*t) +0.9$ | SRSF5 Speckles |
| 0.7486 | 0.2514 | $f(t)=-0.231*exp(-0.114*t) + -0.569*exp(-0.82*t) +0.919$ | SRSF5 Transcription site |
| 0.7852 | 0.2148 | $f(t)=-0.194*exp(-0.0765*t) + -0.423*exp(-1.18*t) +0.903$ | U2AF65 Nucleoplasm |
| 0.7508 | 0.2492 | $f(t)=-0.224*exp(-0.0823*t) + -0.448*exp(-1.52*t) +0.899$ | U2AF65 Speckles |
| 0.7310 | 0.2690 | $f(t)=-0.241*exp(-0.0752*t) + -0.403*exp(-1.11*t) +0.896$ | U2AF65 Transcription site |
| 0.6938 | 0.3062 | $f(t)=-0.293*exp(-0.0498*t) + -0.509*exp(-0.264*t) +0.957$ | U1-70K Nucleoplasm |
| 0.6515 | 0.3485 | $f(t)=-0.315*exp(-0.0407*t) + -0.416*exp(-0.465*t) +0.904$ | U1-70K speckle |
| 0.5592 | 0.4408 | $f(t)=-0.398*exp(-0.0403*t) + -0.386*exp(-0.345*t) +0.903$ | U170K Transcription site |
| 0.6938 | 0.3062 | $f(t)=-0.293*exp(-0.0499*t) + -0.509*exp(-0.264*t) +0.957$ | SRSF7 Nucleoplasm |
| 0.7247 | 0.2753 | $f(t)=-0.258*exp(-0.0286*t) + -0.529*exp(-0.152*t) +0.937$ | SRSF7 Speckle |
| 0.5815 | 0.4185 | $f(t)=-0.408*exp(-0.0364*t) + -0.508*exp(-0.159*t) +0.975$ | SRSF7 Transcription site |
