# Supplementary material for: Availability of splicing factors in the nucleoplasm can regulate the release of mRNA from the gene after transcription
Source: PLoS Genet. 2019 Nov 25;15(11):e1008459. doi: 10.1371/journal.pgen.1008459 (PMC6901260; doi:10.1371/journal.pgen.1008459)
Supplement: S3 Table — (DOCX) [file pgen.1008459.s011.docx]

| **Splicing factor** | **P-value** |
| --- | --- |
| SRSF1 | 0.7671 |
| SRSF2 | <0.0001 |
| SRSF3 | 0.9202 |
| SRSF4 | <0.0001 |
| SRSF5 | 0.3199 |
| SRSF6 | <0.0001 |
| SRSF7 | <0.0001 |
