# Supplementary material for: Availability of splicing factors in the nucleoplasm can regulate the release of mRNA from the gene after transcription
Source: PLoS Genet. 2019 Nov 25;15(11):e1008459. doi: 10.1371/journal.pgen.1008459 (PMC6901260; doi:10.1371/journal.pgen.1008459)
Supplement: S4 Table — (DOCX) [file pgen.1008459.s012.docx]

| **Splicing factor** | **Region** | **P-value** |
| --- | --- | --- |
| SRSF7 | Transcription site | 0.0027 |
| SRSF7 | Nucleoplasm | 0.0147 |
| PRP8 | Transcription site | 0.0702 |
| PRP8 | Nucleoplasm | 0.0089 |
| SRSF2 | Transcription site | 0.7252 |
| SRSF2 | Nucleoplasm | 0.5629 |
| U1-70K | Transcription site | 0.2252 |
| U1-70K | Nucleoplasm | 0.0089 |
