# Supplementary material for: Availability of splicing factors in the nucleoplasm can regulate the release of mRNA from the gene after transcription
Source: PLoS Genet. 2019 Nov 25;15(11):e1008459. doi: 10.1371/journal.pgen.1008459 (PMC6901260; doi:10.1371/journal.pgen.1008459)
Supplement: S5 Table — (DOCX) [file pgen.1008459.s013.docx]

| **Restriction enzyme** | **Primer reverse** | **Primer forward** | **Gene** |
| --- | --- | --- | --- |
| HindIII, KpnI | TATGGTACCGTCGACTGCAGTTAGGTACGA | ATAAGCTTGCATGTCGGGAGGTGGTGTGAT | SRSF1 |
| HindIII, KpnI | ATAGGTACCTTAAGAGGACACCGCTCCTTC | ATAAAGCTTATATGAGCTACGGCCGCCCCC | SRSF2 |
| KpnI, BamHI | ATAGGATCCCTATTTCCTTTCATTTGACCT | ATAGGTACCATGCATCGTGATTCCTGTCCA | SRSF3 |
| KpnI, BamHI | ATAGGATCCATGTAGGACCTTGAGTGGGACCTAGAT | ATAGGTACCATGCCGCGGGTGTACATCGGCCGCC | SRSF4 |
| HindIII, KpnI | ATAGGTACCTTAATTGCCACTGTCAACTGA | ATAAAGCTTGCATGAGTGGCTGTCGGGTAT | SRSF5 |
| HindIII, KpnI | ATAGGTACCTTAATCTCTGGAACTCGACCT | ATAAAGCTTGTATGCCGCGCGTCTACATAG | SRSF6 |
| KpnI, BamHI | ATAGGATCCTCAGTCCATTCTTTCAGGACT | ATAGGTACCATGTCGCGTTACGGGCGGTAC | SRSF7 |
| KpnI, BamHI | ATAGGATCCGAACCTGGCTTGTCTTCAACTA | ATAGGTACCATGCCGCGGGTGTACATCGGCCGCC | SRSF4 without RS |
| BsrGI,  EcoRI | ATAGAATTCTACGGGGCCCATCAACTTTAA | ATATGTACAGAGCTTGCATGTCGGGAGGTG | SRSF1 without RS |
| EcoRI,  BamHI | TATGGATCCATGTAGGACCTTGAGTGGGAC | ATAGAATTCCCAGACGACGCCGGTCCTACT | SRSF4 RS domain |
| EcoRI, BamHI | TATGGATCCTTAATCTCTGGAACTCGACCT | ATAGAATTCACACAAGCCATAGGCGATCTT | SRSF6 RS domain |
| BamHI, NheI | ATAGCTAGCTCACGTATGCTTTTTAAGTGG | ATAGGATCCATGGTGAGCAAGGGCGAGGAG | Clk1 |
| HindIII, KpnI | ATAGGTACCCTATCGAAACAACCTGGTGAA | ATAAAGCTTGCAACATTTGCTCTGTCTGCC | TNPO3 |
| HindIII, KpnI | TATGGTACCTTAGGAGTTAAGCCAAGGGTG | ATAAAGCTTGCATGGAGCGGAAAGTGCTTG | SRPK1 |
