# Supplementary material for: Availability of splicing factors in the nucleoplasm can regulate the release of mRNA from the gene after transcription
Source: PLoS Genet. 2019 Nov 25;15(11):e1008459. doi: 10.1371/journal.pgen.1008459 (PMC6901260; doi:10.1371/journal.pgen.1008459)
Supplement: S6 Table — Calculated for different splicing efficiency values. Normalized by E3 control data. (DOCX) [file pgen.1008459.s014.docx]

|  |  |  |  |  | **Splicing efficiency values** | | | |
| --- | --- | --- | --- | --- | --- | --- | --- | --- |
|  | **Retention parameters** | **Splicing efficiency** | **intron/exon** |  | **500.0** | **750.0** | **1000.0** | **1500.0** |
| e3 | 0.020 | 200.000 | 0.059 |  | 0.410 | 0.268 | 0.202 | 0.136 |
| e3 | 0.020 | 500.000 | 0.144 |  | 1.000 | 0.655 | 0.493 | 0.331 |
| e3 | 0.020 | 750.000 | 0.220 |  | 1.528 | 1.000 | 0.753 | 0.506 |
| e3 | 0.020 | 1000.000 | 0.292 |  | 2.028 | 1.327 | 1.000 | 0.671 |
| e3 | 0.020 | 1500.000 | 0.435 |  | 3.021 | 1.977 | 1.490 | 1.000 |
| e3 | 0.020 | 2000.000 | 0.581 |  | 4.035 | 2.641 | 1.990 | 1.336 |
| e3 | 0.020 | 2500.000 | 0.633 |  | 4.396 | 2.877 | 2.168 | 1.455 |
| e3 | 0.020 | NaN (5000000000000) | 0.651 |  | 4.521 | 2.959 | 2.229 | 1.497 |
|  |  |  |  |  | 0.000 | 0.000 | 0.000 | 0.000 |
|  |  |  |  |  | 0.000 | 0.000 | 0.000 | 0.000 |
| e6 | 0.020 | 200.000 | 0.126 |  | 0.875 | 0.573 | 0.432 | 0.290 |
| e6 | 0.020 | 500.000 | 0.318 |  | 2.208 | 1.445 | 1.089 | 0.731 |
| e6 | 0.020 | 750.000 | 0.479 |  | 3.326 | 2.177 | 1.640 | 1.101 |
| e6 | 0.020 | 1000.000 | 0.639 |  | 4.438 | 2.905 | 2.188 | 1.469 |
| e6 | 0.020 | 1500.000 | 0.955 |  | 6.632 | 4.341 | 3.271 | 2.195 |
| e6 | 0.020 | 2500.000 | 1.520 |  | 10.556 | 6.909 | 5.205 | 3.494 |
| e6 | 0.020 | NaN (5000000000000) | 2.290 |  | 15.903 | 10.409 | 7.842 | 5.264 |
|  |  |  |  |  | 0.000 | 0.000 | 0.000 | 0.000 |
|  |  |  |  |  | 0.000 | 0.000 | 0.000 | 0.000 |
| e6 | 0.002 | 200.000 | 0.091 |  | 0.634 | 0.415 | 0.313 | 0.210 |
| e6 | 0.002 | 500.000 | 0.229 |  | 1.590 | 1.041 | 0.784 | 0.526 |
| e6 | 0.002 | 750.000 | 0.343 |  | 2.382 | 1.559 | 1.175 | 0.789 |
| e6 | 0.002 | 1000.000 | 0.455 |  | 3.160 | 2.068 | 1.558 | 1.046 |
| e6 | 0.002 | 1500.000 | 0.682 |  | 4.736 | 3.100 | 2.336 | 1.568 |
| e6 | 0.002 | 2000.000 | 0.906 |  | 6.292 | 4.118 | 3.103 | 2.083 |
| e6 | 0.002 | 2500.000 | 1.110 |  | 7.708 | 5.045 | 3.801 | 2.552 |
| e6 | 0.002 | NaN (5000000000000) | 2.780 |  | 19.306 | 12.636 | 9.521 | 6.391 |
